# Shielded environments reduce stress in alien Asteraceae species during hot and dry summers along urban‐to‐rural gradients

**DOI:** 10.1002/ece3.7872

**Published:** 2021-07-13

**Authors:** Charly Géron, Jonas J. Lembrechts, Ivan Nijs, Arnaud Monty

**Affiliations:** ^1^ Biodiversity and Landscape TERRA Research Centre Gembloux Agro‐Bio Tech University of Liège Gembloux Belgium; ^2^ Plants and Ecosystems University of Antwerp Wilrijk Belgium

**Keywords:** Alien plant species, phenotypic response, sky view factor, urbanity, urbanization

## Abstract

Urban environments often host a greater abundance and diversity of alien plant species than rural areas. This is frequently linked to higher disturbance and propagule pressure, but could also be related to the additional establishment of species from warmer native ranges in cities, facilitated by the latter's higher air temperatures and drier soils. A hitherto unresolved question is how stressful the urban environments become during climate extremes such as heatwaves and droughts. Do such episodes still favor alien plant species, or set them back? We used in situ measured phenotypic leaf and development trait responses of the six most widespread alien Asteraceae species from various native climates along Belgian urban‐to‐rural gradients, measured during two unusually warm and dry summers. Urbanization was characterized by three factors: the percentage of artificially sealed surfaces (urbanity, measured at three spatial scales from in situ to satellite‐based), the vegetation cover and the sky view factor (SVF, fraction of the hemisphere not blocked by buildings or vegetation). Across species, either from colder or warmer native climates, we found a predominant protective effect of shielded environments that block solar radiation (low SVF) along the entire urban‐to‐rural gradient. Such environments induced lower leaf anthocyanins and flavonols indices, indicating heat stress mitigation. Shielded environments also increased specific leaf area (SLA), a typical shade response. We found that vegetated areas had a secondary importance, increasing the chlorophyll content and decreasing the flavonols index, but these effects were not consistent across species. Finally, urbanity at the organism spatial scale decreased plant height, while broader‐scale urbanity had no significant influence. Our results suggest that sealed surfaces constrain alien Asteraceae during unusually warm and dry summers, while shielded environments protect them, possibly canceling out the lack of light. These findings shed new light on alien plant species success along urban‐to‐rural gradients in a changing climate.

## Graphical abstract

Sealed surfaces constrain alien Asteraceae during unusually warm and dry summers, while shielded environments protect them, possibly cancelling out the lack of light. These findings shed new light on alien plant species success along urban‐to‐rural gradients in a changing climate.

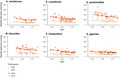

## 1 INTRODUCTION

Urbanization currently affects the whole world, and about 70% of the population is expected to live in urban areas by 2050 (Gross, 2016; Terama et al., 2019). With already more than 74% of its population living in cities today, Europe is one of the most urbanized regions in the world and displays the highest road and rail density (Hulme, 2009). Urban environments are made of a complex assemblage of different land covers directly related to their multiple uses (Ward et al., 2016). They thus consist of a patchwork of microhabitats with a wide range of growing conditions for plant species and are widely recognized as plant diverse (Schmidt et al., 2014). However, urbanization promotes the loss of native plant species and their replacement by alien species (McKinney, 2006). This phenomenon can be explained by two factors: a high alien propagule pressure due to a dense transport infrastructure network and alien plant cultivation in gardens and parks, and high level of anthropogenic disturbance on top of the aforementioned high habitat heterogeneity (Čeplová et al., 2017; Štajerová et al., 2017). Cities can then act as alien plant sources for the rural environments, with the spread of propagules along urban‐to‐rural gradients being facilitated by corridors such as roads and rivers (Botham et al., 2009).

Urban environments are well known for their modified microclimatic conditions compared to rural areas. This can be explained in part by the relationship between urban morphology and solar irradiance, affecting air temperature (Dirksen et al., 2019). The low albedo of urban structures, feeble evapotranspiration due to decreased vegetation cover, heat release via anthropogenic activities as well as the heat storage from buildings, all together lead to higher temperatures in cities than in the rural surroundings (urban heat island (UHI) effects (Oke, 1981; Theeuwes et al., 2017)). The percentage of impervious surfaces which considers the urban sprawl (hereafter termed “urbanity”) is positively related to the intensity of UHI effects with stronger observed UHIs in the most impervious areas (Oke, 1981; Ward et al., 2016). Importantly, the effects of urbanity on temperature variations depend on the scale considered, and these effects can interact. As such, while the UHI effects operate at the landscape scale, a broad range of cooler and warmer microhabitats occurs at the scale of a few meters due to the variety of land uses in the urban environment (Ren et al., 2013). The UHI effects are also variable during the day and are typically higher during calm summer evenings and nights in western Europe (Top et al., 2020). The daily maximum UHI intensity between the city center and rural countryside of Ghent in Belgium in summer equals 2.5°C on average, with peaks up to 6°C (Caluwaerts et al., 2020).

The urban morphology is complex and can be described by the sky view factor (SVF) which takes into account the urban geometry and building density (Middel et al., 2018). The SVF measures the proportion of open sky at a given location, which is a proxy for the ratio of the radiation received by a surface from the sky to the radiation emitted (Tan et al., 2016; Watson & Johnson, 1987). The height, length, and spacing of the buildings affect the energy fluxes and result in highly heterogeneous urban habitats in terms of micro‐scale temperature variation (Grimmond et al., 2001). These intra‐urban temperature differences are highly linked to the SVF (Chen et al., 2012). UHI effects are locally enhanced due to trapped solar and anthropogenic energy in urban canyons characterized by dense building patterns (Bonamente et al., 2013; Theeuwes et al., 2017). As a result, built‐up areas with low SVF cool down less efficiently and have diminished temperature variations than rural environments (Oke, 1981). Although overheating due to solar radiation can be reduced in areas with very low SFV, these locations also have reduced airflow and higher solar reflection, which increases heat trapping (Steeneveld et al., 2011). Another factor that significantly impacts both the intra‐urban microclimate and the temperature differences between rural and urban environments is the amount of vegetated surfaces (Tan et al., 2016). Even if trees and parks also induce low SVF values, they limit heat storage and mitigate UHI effects through evapotranspiration, especially when irrigated (Dirksen et al., 2019). Local‐scale temperature variations within urban areas are thus influenced by the combination of 3‐D urban geometry and vegetation, which can be observed in vegetated urban canyons that experience cooler temperatures than nonvegetated ones (Caluwaerts et al., 2020; Steeneveld et al., 2011). Intra‐urban temperature variations also interact with those at the landscape scale between urban and rural environments due to UHI effects (Guo et al., 2015; Kaiser et al., 2016).

Plants respond to the environment by shifting their phenotype (Nicotra et al., 2010; Rivkin et al., 2019), linked to fitness (Piana et al., 2019). However, most studies about alien plant species in urban environments have neglected within‐species trait plasticity and instead focused on the selection of species‐level traits along urban‐to‐rural gradients or have used spatiotemporal scales that might be too coarse to detect phenotypic responses (Williams et al., 2015). A notable exception is the study by Zipper et al. (2017), who found higher UHI‐induced evapotranspirative demand during midsummer in Madison, United States of America. Such studies are rare, and little is known about how rural versus urban and intra‐urban microhabitat differences influence alien plant species phenotypic responses, especially during heatwaves and droughts which are predicted to be more common and severe under climate change in general, and in particular in urban environments (Hamdi et al., 2015; Rosenzweig et al., 2018).

The Asteraceae family is one of the plant families with the highest number of alien species across the world (Daehler, 1998). We studied the phenotypic response of the six most widespread Asteraceae alien plant species along urban‐to‐rural gradients in the Atlantic biogeographical region of Belgium (European Environment Agency (EEA), 2011) during two unusually dry and warm summers, focusing on leaf pigments and specific leaf area (SLA) and developmental (height, internode space) variables. We assessed urbanization and its effects on microhabitat conditions at three spatial scales using in situ measured and satellite‐based urbanity proxies, a remotely sensed proxy of the vegetation cover in the surroundings of the studied plants, and the SVF measured at the level of the studied plants. The following questions are addressed: (i) Do alien plant species show phenotypical variation along urban‐to‐rural gradients, and at which scales? (ii) Do the urbanization proxies, that is, the urbanity at different scales, the vegetation cover and the SVF, interact in determining these phenotypic responses? (iii) Are urbanization effects on phenotypic responses modulated by the vegetation cover in the alien plants’ surroundings?

## 2 METHOD

### 2.1 Study area

We performed our study in the Atlantic biogeographical region of Belgium (European Environment Agency (EEA), 2011), that is the area north of the Meuse river. This spatial delineation was chosen in order to consider a study region with background climatic conditions as homogeneous as possible. The area encompassed Flanders, Brussels Capital Region, and the north of Wallonia (Appendix S1), which contain some of the most urbanized parts of Belgium (urbanity in Flanders, for example, equaled 16% in 2015, De Ridder et al., 2015) but also rural zones.

### 2.2 Species selection

We selected the six most common alien plant species from the Asteraceae family in Belgium (http://www.q‐bank.eu/Plants/, Belgian Flora: (Lambinon et al., 2012)), origin between brackets: *Artemisia verlotiorum* Lamotte, hemicryptophyte, 1–1.5 m (China); *Erigeron canadensis* L., therophyte, 0.8–1 m (North America); *Galinsoga quadriradiata* Ruiz & Pav., therophyte, 0.1–0.7 m (Central America); *Matricaria discoidea* DC., therophyte, 0.2–0.4 m (North America); *Senecio inaequidens* DC., chamaephyte, 0.5–1.5 m (South Africa); *Solidago gigantea* Aiton, hemicryptophyte, 0.8–1.2 m (North America).

### 2.3 Location selection in Belgium and field campaigns

High‐resolution coordinates (GPS points with a mean precision of 140 meters) of the occurrences of the selected species in the study area were obtained via the citizen science observation platform waarnemingen.be/observations.be (we kept only the occurrences for whom species identifications were verified by experts, Natuurpunt et al., 2018), for the period from 1998 to 2018. The urban‐to‐rural gradients were depicted using remotely sensed urbanity ((European Environment Agency (EEA), 2019), percentage of sealed surfaces, original scale: 400 m²) at two scales: 400 m² (20 × 20 m) and 9 km² (3 × 3 km). The use of these two scales allowed us to take into account scale‐dependent effects of urbanization: UHI effects operate most strongly at the 9 km² resolution, while at 400 m² resolution urban microclimates are captured (Brans et al., 2017; Kaiser et al., 2016). The 400 m² scale will later on be referred as the “local urbanity scale”. The original layer was aggregated at 9 km² using the mean and is hereafter referred to as the “landscape urbanity scale”.

We conducted a random selection of species’ occurrences, yet maximizing the range of urbanity at both the local and landscape scale as well as the range of possible combinations of the two scales. This was performed by dividing the local and landscape urbanity scales in classes of 10% and selecting an equal number of random occurrences from each combination of local and landscape‐level classes, if occupied by the species. This way, we obtained the full, crossed spectrum of the two urbanity scales specific to each considered species (Appendix S1). Each location selected as such was visited and, if the target alien species was found, one representative individual was chosen and its precise location was taken with a handheld GPS. Management actions happen at different intensities along the Belgian urban‐to‐rural gradients, but it was not possible to rule out variation in these actions between our study locations. Therefore, individuals with obvious damages or that have been cut or mown were excluded, as such damages strongly affect plant responses such as growth (Sehrt et al., 2020; Song et al., 2018). This required ten field days in July 2018, from 07/09 to 08/03. The same locations were revisited in summer 2019, for a period of eight field days extending from 06/18 to 07/03. As such, 237 and 250 individuals were visited in 2018 and 2019, respectively: 22 and 34 for *A. verlotiorum*, 53 and 63 for *E. canadensis*, 39 and 33 for *G. quadriradiata*, 35 and 36 for *M. discoidea*, 41 and 38 for *S. inaequidens*, and 47 and 46 for *S. gigantea*. Eighty % of the locations of 2018 could be resurveyed in 2019. This difference in the occurrences between the two field seasons was mainly due to annual species such as *G. quadriradita* or *M. discoidea* not always being found back in the second field season. In that case, a new location was selected with comparable urbanity values. When possible, new locations were added to increase spatial distribution. Special effort was made to add new locations for *A. verlotiorum* as it had the lowest cover in 2018. The two summers in our study were characterized by unusually warm and dry weather compared with the climatic means of the 1981–2010 period, calculated for the climatic reference station for Belgium, Uccle. During the 2018 and 2019 summers (June, July, and August), the mean temperature was respectively 2.3 and 1.6°C higher than normal, precipitation 40% and 11% lower than normal, and total hours of sunshine 120% and 124% above normal (RMI, 2018a, 2019a). The Royal Meteorological Institute of Belgium defines a heatwave as at least five consecutive days with a maximum temperature of 25°C or more, of which at least three days reach 30°C or more (www.meteo.be). As such, our field campaigns took place during two heatwaves in 2018 (from 07/13 to 07/27, and from 07/29 to 08/07, with only three days of precipitation), and one heatwave in 2019 (from 06/23 to 06/30, with no precipitation).

### 2.4 Field measurements

#### 2.4.1 Environmental variables

To quantify the growing conditions of each alien plant individual, we used (1) a proxy for urbanity at 3 spatial scales, (2) the sky view factor (SVF), and (3) the vegetation cover at a scale of 400 m².

In addition to the local and landscape scale urbanity referred to above, we acquired the fine‐scale urbanity of the growing environment of the studied alien plant individuals by estimating the percentage of sealed surface in a 40 × 40 cm square centered on the focal organism. This variable will be referred to as the “organism urbanity scale.”

The SVF relies on the calculation of the proportion of the sky that is open at a certain location, which can be obtained from analyses of fish eye photography, digital elevation models, or obstacle angles (Johnson & Watson, 1984; Middel et al., 2018). Using a clinometer, we measured the standardized horizon angle (taken at 1 m height) to which the sky was visible in each cardinal direction, without distinguishing between buildings or vegetation: $\mathrm{SVF}=\frac{\left((90-N)+(90-E)+(90-S)+(90-W)\right)}{360}$, with N, E, S, W, standardized horizon angle toward north, east, south, and west, at the location of each studied plant. The SVF is directly linked to the solar energy which a place receives due to the local 3‐D geometry. As such, an open terrain gets a higher amount of short and long wave radiation (SVF ≈ 1) than a shielded terrain (SVF ≈ 0) (Dirksen et al., 2019; Oke, 1981).

To further describe the nature of the growing environment (human artifacts contributing to urban warming or heat mitigation by vegetation), the vegetation cover at a scale of 400 m² (20 × 20 m, same as the local urbanity scale) was calculated from a pixel‐based classification of the vegetation (Initial scale: 4 m², LifeWatch ‐ FWB project, 2015), while excluding water, built‐up and arable land. This allowed us to pool short and tall vegetation, as both mitigate urban heat island effects through evaporative cooling and/or vegetative shading (Caluwaerts et al., 2020; Wang et al., 2015).

#### 2.4.2 Phenotypic responses

To depict the phenotypic responses of the studied alien species along the urban‐to‐rural gradients, we measured their height and internode space, as well as the following leaf variables: the chlorophyll content, anthocyanins, and flavonols indices and the specific leaf area (SLA), for each individual.

Plant height and internode space respond to drought, heat as well as shading, with a limited development in dry and warm conditions, but elongated internodes and taller plants in shaded areas (Gorton et al., 2018; Nicotra et al., 2010; Yang & Li, 2017). Chlorophyll content, as well as the anthocyanins and flavonols indices inform about light exposure, drought, and temperature extremes, with lower chlorophyll content but higher anthocyanins and flavonols indices in dry, high UV, and warm conditions (Ballizany et al., 2012; Hatier & Gould, 2009). SLA assesses shading impact and resource uptake, with higher values in shielded conditions and high nutrient availability (Gregg et al., 2003; Song et al., 2019). While plant height integrates the growing season, SLA and pigments such as anthocyanins, chlorophyll, or flavonols reflect the response of individuals over a much shorter time period—that of leaf ontogeny and lifespan (Fernández Honaine et al., 2019; Fusaro et al., 2019; Song et al., 2019).

The height of the studied plant, as well as the internode space just below the youngest fully developed leaf, were measured using a folding meter.

The chlorophyll content (µg/cm²) and anthocyanins and flavonols indices were acquired in the field on the three latest developed leaves using the Dualex^®^ Scientific + (Force‐A, Orsay, France) and averaged. The chlorophyll content is obtained via the transmittance ratio at two different wavelengths, one in the far‐red absorbed by chlorophyll and one in the near‐infrared as a reference. The Dualex acquires the anthocyanins and flavonols indices (relative absorption) via the screening effect of these polyphenols on chlorophyll fluorescence. The chlorophyll content as well as the anthocyanins and flavonols indices were not measured for *M. discoidea*, due to incompatible leaf size and shape.

The same leaves as measured above were ultimately collected and processed. The fresh leaf area was measured using a flatbed scanner and later analyzed in ImageJ (Rasband, 2018), and the dry mass was measured after drying the leaves at 60°C for 48 hr. The SLA was calculated following. $\mathrm{SLA}=\frac{\mathrm{leaf}\;\mathrm{area}\;({\mathrm{cm}}^{2})}{\mathrm{dry}\;\mathrm{mass}\;(g)}$

### 2.5 Temperature estimates over the modeled native ranges

In order to get a proxy of the studied alien species native climatic niche, species distribution models were built with their native occurrences from the Global Biodiversity Information Facility (GBIF, 2020), using maximum entropy modeling (Maxent) (Phillips et al., 2004, 2006, 2017). Maxent predictions were transformed into presence cells and constituted the studied species modeled native ranges (see detailed method in Appendix S2, and Maxent model information in Appendix S3). To coarsely depict the native climatic conditions of each species, we extracted the 19 WorldClim bioclimatic predictors (Fick & Hijmans, 2017) with a 5 × 5 km grid over their modeled native ranges. To simplify the set of 19 bioclimatic predictors, we conducted a principal component analysis after standardizing them following the default settings of the R package FactoMineR, in order for each of the variables to have a standard deviation equal to 1 and a mean equal to 0 (PCA, Husson et al., 2020). We then kept the first PCA axis (explaining 47% of variance) which most strongly correlated with the minimum temperature of the coldest month (Bio 6, positively correlated), the mean temperature of the coldest quarter (i.e., the coldest four months of the year, Bio 11, positive), and the annual mean temperature (Bio 1, positive) (Appendix S4). We calculated the coordinates of the 5 × 5 km grid points for each species’ native modeled range along this first PCA axis. To depict their native niche optimum along this axis, the mean was calculated for each species separately and was referred to as “winter temperatures.”

To compare the mean annual temperature conditions in the species modeled native ranges, the average of the mean annual temperature (Bio 1) was calculated: *A*. *verlotiorum*: 18.2°C, *E. canadensis*: 16.7°C; *G. quadriradiata*: 18.6°C; *M. discoidea*: 6.7°C; *S. inaequidens*: 17.1°C; and *S. gigantea*: 7.2°C. The mean annual temperature in the study area was 10.2°C. Two thirds of the studied species thus originated from warmer native ranges than the study area in terms of mean annual temperature.

### 2.6 Statistical analyses

Spearman correlations were performed to test for the relationships between the organism, local and landscape urbanity, the vegetation cover at 400 m², and the SVF.

We verified potential effects of the date of observation within each field season (covering a total period of 26 days in 2018 and of 16 days in 2019) on the alien plant phenotypic response with linear mixed models for each of the developmental (height and internode space) and leaf variables (SLA, pigments: chlorophyll content, anthocyanins, and flavonols indices) (R package lme4, Bates et al., 2020, with species identity as a random intercept). This was found to have none or limited influence on the developmental and leaf variables and was therefore not included in the next models.

The relationships between the developmental and leaf variables (as response variables) and the urbanity at three scales (organism, local, and landscape), the SVF, the vegetation cover, the winter temperatures, and the field season (explanatory variables) were analyzed using linear mixed models (across all species, with species identity as a random intercept) (R package lme4, Bates et al., 2020), and linear models (species‐specific models, but without the winter temperatures) (R package Stats, R Core Team & contributors worldwide, 2020). We verified if the addition of the species identity as a random intercept increased the fit of the across‐species linear mixed models for each of the response variables. For this, we compared the Akaike information criterion (AIC) between the full models with and without this random intercept (Galwey, 2006). For all response variables, the addition of the species identity as a random intercept decreased the AIC with more than two units and significantly increased the fit of the across‐species models. To meet residual normality, the flavonols index, height, internode space, and SLA were log‐transformed, an arcsine transformation was applied to the anthocyanins index, and a square root transformation was applied to chlorophyll content.

The full models included all two‐way interactions of the explanatory variables. Quadratic terms for the individual, local and landscape urbanity scales, the vegetation cover and the SVF were included to test for an optimum in conditions along the gradient (Williams et al., 2015). Only the inclusion of the quadratic term of the SVF was retained in the full models and significant (*p* < .05). Consequently, interactions between the SVF² and each of the other explanatory variable were included in the full models. The variance inflation factors (VIF) were checked for the explanatory variables in the full models (excluding interactions or quadratic effects, Lüdecke et al., 2020). This revealed no multicollinearity in the full models (1 < VIF < 1.80). We used the *dredge* function on the full models (R package MuMIn, Barton, 2019) to select the best models, that is, those with a ΔAIC < 2 (a difference of less than two in the AIC, in comparison with the model with the lowest AIC). We then performed model averaging on these best models (function *model.avg*, MuMIn, K. Barton, 2009). Indeed, large model sets may not contain a single best model and some models may differ in their data fit by only small amounts. In these cases, model averaging allows to account for model selection uncertainty and to obtain robust parameter estimates or predictions (Grueber et al., 2011). For each of the response variables, we calculated the pseudo‐R‐squared for the across‐species models with the lowest AIC (R package MuMIn, K. Barton, 2009). Additionally, we follow the reasoning that relationships displaying significance below *p* = .05 by chance would occur for five percent of the models or tests (McDonald, 2014). In our case, we found statistically very significant or significant effects (with *p* values below .001 or .01, respectively) of the explanatory variables on our response variables in five out of six (83%) across‐species averaged models, and in 21 out of 33 (64%) species‐specific averaged models, which is more than the 5% one would expect based on random noise (see Table 1, and Appendix S6, Tables S1–S6; McDonald, 2014).

Finally, for each of the response variable, the across‐species model with the lowest AIC was tested with DHARMa Moran's I tests for potential spatial autocorrelation of the residuals. These test results were found not to be significant (Hartig & Lohse, 2020).

ArcMap 10.5.1 (ArcGis Desktop, 2017) was used for spatial processing. All statistical analyses were performed in R, version 3.5.2 (R Core Team, 2018), and *p* = .05 was taken as threshold for significance.

## 3 RESULTS

### 3.1 Across‐species models

Across all species and both field seasons, the SVF and the vegetation cover overruled urbanity (at the organism, local, or landscape scale) as most important drivers of alien plant species phenotypic trait variations. In the across‐species models, the SVF and the vegetation cover were retained in 83% and 67% of the averaged models, respectively (Table 1, significant in 50% and 33% of the best models, respectively). The urbanity at the organism scale was the only urbanity level retained in the averaged models (Table 1, retained in 16% of the averaged models, and significant in 16% of the best models).

**TABLE 1:** Estimates, standard errors (between brackets) for the averaged models testing for the drivers of chlorophyll content, flavonols and anthocyanins indices, the specific leaf area (SLA), the internode space, and the plant height across all species

	Chlorophyll content	Flavonols index	Anthocyanins index	Specific leaf area (SLA)	Internode space	Plant height
(Intercept)	4.77^***(***)^ (0.17)	0.35^*(***)^ (0.17)	0.11^(***)^ (0.06)	6.08^***(***)^ (0.28)	0.80^(*)^ (0.51)	3.88^***(***)^ (0.52)
Urb. organism						−0.01^***(***)^ (<0.001)
SVF		0.63^(**)^ (0.46)	0.15^(**)^ (0.20)	−1.51^(*)^ (0.96)	−*1.08* (1.35)	*0.71* (1.43)
SVF^2		−0.23 (0.39)	−0.02 (0.17)	*0.58* (0.85)	*0.65* (1.12)	−*0.95* (1.24)
Vegetation cover	0.40^*(*)^ (0.18)	−0.40^**(**)^ (0.13)		−0.02 (0.07)		0.05 (0.23)
Field season 2019	−*0.09* (0.12)	−0.18^***(***)^ (0.02)	0.08^***(***)^ (0.01)	−0.93^***(***)^ (0.09)	−0.58^*(**)^ (0.27)	−0.47^*(***)^ (0.22)
Winter temperatures	0.03 (0.06)	−0.14^**(**)^ (0.05)				
SVF × Vegetation cover		0.57^*(**)^ (0.22)				−0.19 (0.47)
SVF × Field season 2019					*0.29* (0.44)	0.17 (0.35)
SVF × Winter temperatures		0.18^***(***)^ (0.03)				
Vegetation cover × Field season 2019	−0.82^***(***)^ (0.23)			0.16 (0.18)		
Num. obs.	347	347	345	456	473	473
Model with the lowest AIC information						
Pseudo‐*R*² conditional	0.25	0.64	0.24	0.56	0.59	0.62
Pseudo‐*R*² marginal	0.10	0.27	0.10	0.43	0.04	0.06

Note

Significant effects are in bold and coded as follows: ***p < .001; **p < .01; *p < .05. Numbers in italic correspond to the variables or interactions present in the model with the lowest AIC, and significance codes between brackets correspond to significance in the model with the lowest AIC. Blank cells correspond to explanatory variables or interactions not present in the averaged model. All two‐way interactions were tested, yet those that were retained in none of the averaged models are not included in the table. Urb. organism = the percentage of impervious surfaces (urbanity) at the organism scale (40 × 40 cm). The conditional pseudo‐*R*² corresponds to the variance explained by the entire mixed model whereas the marginal pseudo‐*R*² corresponds to the variance explained by the fixed effects only.

**FIGURE 1:**
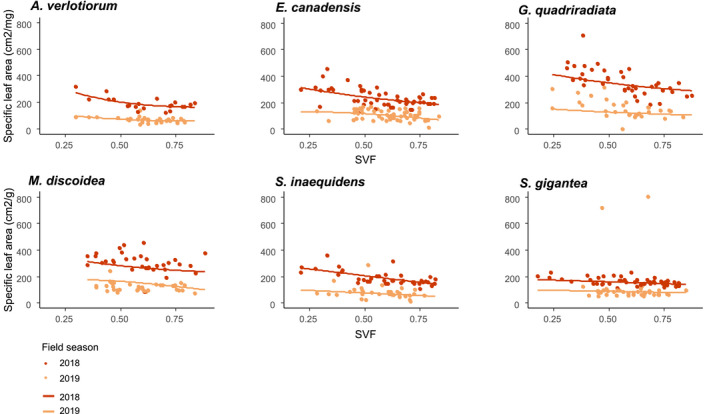
Specific leaf area as a function of the sky view factor (SVF, measured at the level of the individual), for the alien Asteraceae species. Measured values (dots) and modeled trends (lines) reported for 2018 (red) and 2019 (orange) separately, from the species‐specific averaged models (see Appendix S6, Table S1)

**FIGURE 2:**
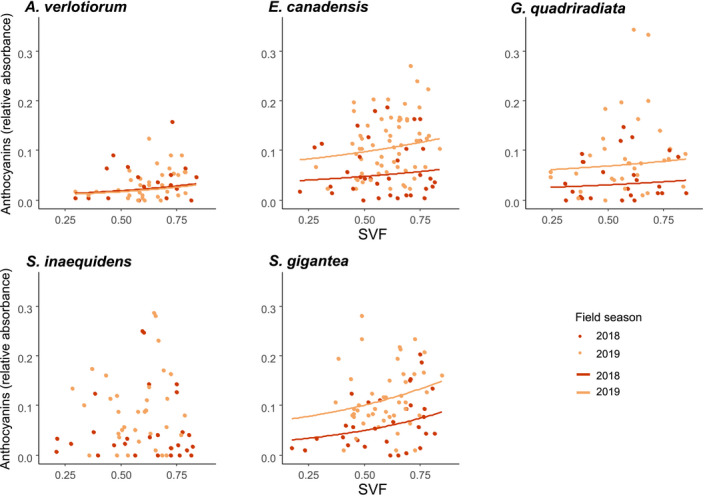
Anthocyanins index as a function of the sky view factor (SVF, measured at the level of the individual), for the alien Asteraceae species. Measured values (dots) and modeled trends (lines) reported for 2018 (red) and 2019 (orange) separately, from the species‐specific averaged models (see Appendix S6, Table S2). If the SVF was not included in the species‐specific averaged models, no trendlines were drawn

**FIGURE 3:**
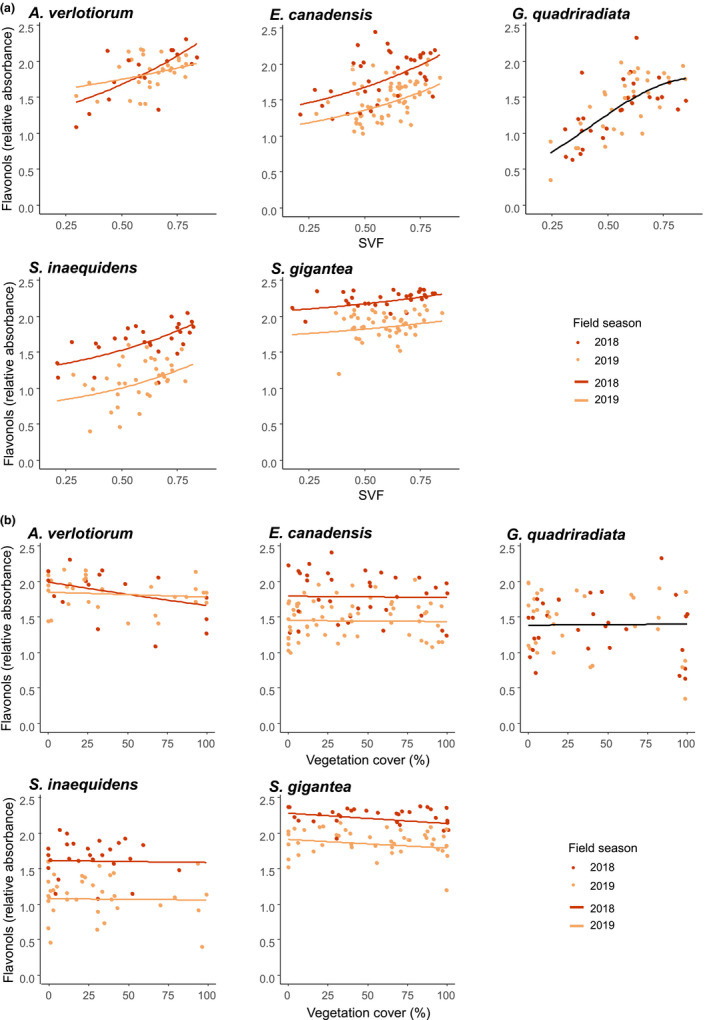
(a) Flavonols index as a function of the sky view factor (SVF, measured at the level of the individual), for the alien Asteraceae species. (b) Flavonols index as a function of the vegetation cover (%, measured at a 400 m² resolution), for the alien Asteraceae species. Measured values (dots) and modeled trends (lines) reported for 2018 (red) and 2019 (orange) separately, or together when no field season effect was found (black), from the species‐specific averaged models (see Appendix S6, Table S3)

We observed increases in anthocyanins and flavonols indices and a decrease in SLA for high SVF values (significant in the best models), while positive effects (nonsignificant) of an increasing SVF on height, and negative (nonsignificant) effects on internode space were also retained in the averaged models (Table 1). The effects of the SVF were largely consistent across species and field seasons (Figures 1, 2, 3a), Appendix S6: Tables S1–S6), even though baseline values differed significantly between field seasons (significant field season effect, yet few or no significant interactions between the SVF and the field season in the averaged models).

The chlorophyll content increased and the flavonols index decreased with increasing vegetation cover (significant in the best models), while a positive and a negative—yet nonsignificant—effect of an increasing vegetation cover on plant height and the SLA, respectively, was retained in the averaged models. However, the effects of the vegetation cover were often not consistent across species (Figure 3b), Appendix S6: Figure S1, Tables S1–S6). Only for the flavonols index, a significant interaction between the SVF and the vegetation cover was found, for which we observed an increase for high values of both the SVF and the vegetation cover (Table 1).

**FIGURE 4:**
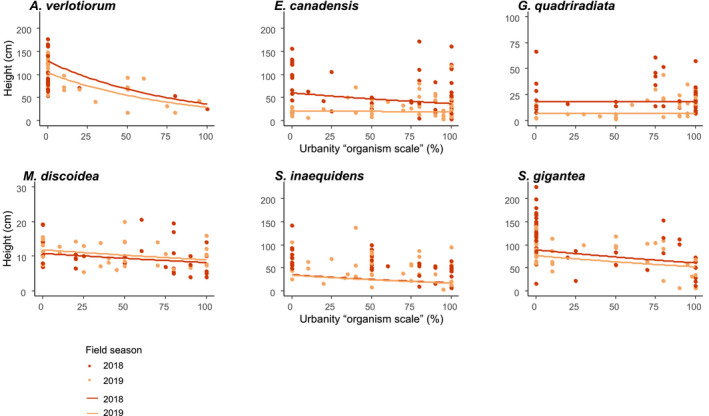
Plant height as a function of urbanity at the “organism scale” (%, measured in a 40 × 40 cm square around the studied individual), for the alien Asteraceae species. Measured values (dots) and modeled trends (lines) reported for 2018 (red) and 2019 (orange) separately, from the species‐specific averaged models (see Appendix S6, Table S4)

Even though a quadratic effect of the SVF was retained in all but one averaged models across all species, it was never significant (Table 1), and for all but one species, linear trends without an optimum along the SVF gradient were observed (Figures 1, 2, 3, 4). Finally, the urbanity variables were not retained in any of the averaged models across all species, except for a significant decrease in plant height with increasing levels of urbanity at the organism scale (Figure 4, Table 1).

### 3.2 Species‐specific models

Species‐specific models showed similar trends, with the SVF and the vegetation cover retained in 85% of the averaged models, yet with the SVF significant in 33% of the best models and the vegetation cover significant in 9% of the best models, respectively (Appendix S6: Tables S1–S6). The urbanity at the landscape and at the local scales showed up in several of the species‐specific models, yet there was a tendency toward higher importance of the urbanity at the organism scale than at the two other scales (Appendix S6: Tables S1–S6). Organism scale urbanity was retained in 82% of the species‐specific averaged models (significant in 36% of the best models), while local and landscape scale urbanity were retained in respectively 67% and 61% of those (significant in 12% and 18% of the best models, respectively). Higher values of the winter temperatures variable (i.e., a warmer climate of origin) led to a higher chlorophyll content and lower flavonols index (although the latter in interaction with the SVF led to higher flavonols index, Table 1).

Interactions between the three urbanity scales or between the three urbanity scales and the SVF were found only in a limited number of cases and species, underlying their relatively low importance. For example, *A. verlotiorum* grew significantly taller at high urbanity at the organism scale and in open areas (high SVF values, Appendix S6: Table S4). Surprisingly, *S. inaequidens* significantly had a lower anthocyanins index if growing either in high local or landscape urbanity, but this decrease of anthocyanins index was reversed for the interaction of local and landscape urbanity (Appendix S6: Table S2). We found contrasting effect of urbanity scales in *A. verlotiorum* which had a significantly higher flavonols index if growing in urbanized local areas, but a lower flavonols index in urbanized landscape environments (Appendix S6: Table S3). Curiously, *G. quadriradiata* and *S. gigantea* were the only studied species that had a lower flavonols index when growing in conditions with high urbanity at the organism scale (and also at the local scale for *G. quadriradiata*). Significant effects of the interaction between the three urbanity scales, the vegetation cover and field season were very limited (Appendix S6: Table S4).

We did not find consistent patterns among the studied species for the chlorophyll content in the species‐specific models, which was not globally significantly related to any of the urbanity variables, the SVF or the vegetation cover (Appendix S6: Figure S1, Table S5). Similarly, we did not find overall patterns for the internode space in the species‐specific models. Smaller internode space was found for two thirds of the studied species in high organism urbanity (but only significant for two species), and no global effects of the SVF or of the vegetation cover were detected (Appendix S6: Figure S2, Table S6).

### 3.3 Data structure

The vegetation cover was significantly negatively correlated with the urbanity at the organism, local, and landscape scales, and the urbanity scales were significantly positively correlated between each other (Appendix S5). The day of observation in each of the field seasons had no significant effect on the response variables except for the anthocyanins and flavonols indices and only in 2019 (respectively, *p* = .05 and *p* = .001), which were lower later in the 2019 field season. Field season itself had a strong significant effect on all the studied variables, with lower chlorophyll content, flavonols index, SLA, height, and internode space in 2019 than in 2018, but higher anthocyanins index (Table 1).

## 4 DISCUSSION

With our analyses of alien Asteraceae phenotypic trait variations during two consecutive hot and dry summers in Belgium, we showed that, across all species, the SVF and the vegetation cover were the prevalent drivers of phenotypic variability in such conditions, and not the organism, local, or landscape urbanity. Alien plants that grew in more shielded environments (low SVF) had lower anthocyanins and flavonols indices, indicating less exposure to UV radiation, drought, and extreme temperatures (Ballizany et al., 2012; Dongyun et al., 2014; Hatier & Gould, 2009; Misyura et al., 2012), whereas they had increased SLA, a typical shade response (Fernández Honaine et al., 2019; Quero et al., 2006). These findings highlight the importance of the protecting microhabitat created by both buildings and vegetation, moderating alien plant stress in unusually warm and dry summer periods (RMI, 2018a, 2019a). Importantly, very few significant interactions between the SVF and urbanity (at any scale) were observed in our study, indicating that shielded environments affected alien plant leaf variables similarly along the urban‐to‐rural gradients. We did not detect compensatory adaptive growth in the species‐specific models, with taller plants or longer internode spaces in shielded environments. On the other hand, the effects of the vegetation cover on the alien plant species phenotypic responses were not very consistent. Although we observed higher chlorophyll and a lower flavonols index in more vegetated situations, indicating less stressful growing condition, this was not verified for each species separately. Moreover, the overall lack of effect of the interaction between the SVF and the vegetation cover suggests that the effect of the SVF as observed was consistent between both nonvegetated and vegetated shielded environments, in spite of the latter's moderating influence on UHI effects and intra‐urban temperature differences under close to normal climatic conditions. Possibly, in our case, vegetation patches dried out during the warmer and drier summer conditions and might have lost their cooling effect (Ward et al., 2016).

Alien plant phenotypic responses across all species did not strongly correlate with urbanity at local and landscape scale. Indeed, significant relationships were limited to a negative effect of the organism scale urbanity on the height of the alien plants, in both field seasons and across most species. Half of the studied species also had shorter internodes in highly impervious areas. Although we did not test for soil or moisture variables, the negative effects of high organism urbanity might be due to a limited access to water and soil space in impervious environments, inducing more stressful growing conditions especially in summer (Zipper et al., 2017). We did not find significant overarching effects of the combination of urbanity scales on alien plant phenotypic responses (but see the higher flavonols and anthoycanins indices in *G. quadriradiata* and *S. inaequidens*, respectively). The lack of consistent relationship between chlorophyll content and urbanization is in line with previous findings, the response of this parameter to drought being for example variable among species (Svensk et al., 2020).

Despite the fact that we found consistent patterns across species, we observed species‐specific relationships which could have been related to their native climatic conditions (Wolf et al., 2020). However, we did not find strong effects of the winter temperatures of the climatic origin of the studied species on phenotypic responses, not even in interaction with the urbanity scales, the SVF, or the vegetation cover. This suggests that all studied species—regardless of their native climate—benefitted from shielded environments and were constrained by sealed surfaces during warm and dry summer periods. Importantly, trends of urbanity and SVF were consistent across both growing seasons. Nevertheless, we observed significant interannual variation, especially in baseline values (model intercepts). For example, flavonols index was globally higher in 2018, in line with the drier and warmer summer compared with 2019. Strikingly, anthocyanins index was higher in 2019, potentially due to very contrasted temperature conditions in spring of that year, with frost in May but a heatwave in June. The greater height and SLA in 2018 than 2019 may have been caused by an earlier start of the growing season (mean April–May–June temperature of 11.5 versus 10.5°C in 2018 and 2019, respectively (RMI, 2019b, RMI, 2018b)). This highlights the importance of interannual studies (Werner et al., 2020).

The environmental variables we considered have all been shown to be representative of the temperature conditions at the landscape or local scales, as the urbanity and the SVF correlate respectively with temperature differences between rural and urban environments, and among intra‐urban microhabitats (Caluwaerts et al., 2020; Ward et al., 2016). However, as mentioned above, there were no large effects of urbanity at local and landscape scales on the phenotypic response of the alien plant species. Possibly, the temperature differences between the extremities of the urban‐to‐rural gradient at those local and landscape urbanity scales represented either too much or too little variation under the unusually warm conditions to be found back in the analyses. As we know that increases of only 1–2°C can already cause higher alien plant species development in urban areas (Song et al., 2012), and differences up to 6°C were observed in summer days between urban and rural places in the study area (Caluwaerts et al., 2020), it is unlikely that thermal variability would have been too small. However, finer‐scaled temperature variation related to the SVF might simply have overshadowed these larger‐scale patterns. Unfortunately, high resolution in situ temperature datasets along urban‐to‐rural gradients are often lacking due to logistical and cost challenges. Future studies would clearly benefit from such knowledge on fine‐scale spatiotemporal variations in microclimatic conditions (Lembrechts et al., 2020).

Urban‐to‐rural gradients represent interesting study systems as they exacerbate climate change effects, such as longer heatwaves (Gorton et al., 2020; Wang et al., 2019). However, studies should consider these areas as complex and investigate them with a combination of variables taking into account the urban sprawl and 3‐D geometry (Tan et al., 2016; Ward et al., 2016). Our results suggest the importance of local microhabitat conditions along urban‐to‐rural gradients for alien plant species, most likely strongly driven by microclimate. Moreover, we found that the location of shielded areas along urban‐to‐rural gradients was not decisive in alien plant phenotypic responses. This suggests that the apparently harsh urban environment may in fact still contain many relatively favorable growing spots. Paved and shadowy urban canyons, as soon as soil space is sufficient for plants establishment, represent warm buffered environments with trapped heat but smaller temperature variations than open sealed urban areas (Theeuwes et al., 2017). These shielded areas would potentially represent less extreme environments in terms of temperature variation, as both open urban and rural areas suffered the most from heat stress during the 2019 warm summer in Belgium, contrary to urban parks or canyons (Top et al., 2020). This is of central importance for urban ecology as such warm and dry summers are predicted to be more common in temperate climates in the future (Kovats et al., 2014). Additionally, even if substrates in urban environments are often covered by anthropogenic materials limiting water evaporation (Kaiser et al., 2016), cities face hydrological droughts due to higher surface water runoff (Pickett & Cadenasso, 2009). However, drought effects on plants depend strongly on the light environment and are less intense in shaded conditions (Quero et al., 2006). The shading in shielded urban areas may therefore have helped counter drought effects in the warm and dry summer conditions of our study.

Most studies on plants along urban‐to‐rural gradients focus on community responses or trait selection by urbanization (see for alien species: Milanović et al., 2020; Wolf et al., 2020; or for native species: Knapp et al., 2008; Song et al., 2019), while studies of the phenotypic responses of alien plant species to urbanization are scarce. Yet, a thorough understanding of alien plant phenotypic responses is essential to comprehend invasions, especially along urban‐to‐rural gradients (Gorton et al., 2018; Monty et al., 2013). Moreover, studies often use urbanization at scales that might be too coarse for the organisms and lead to overlooked trait responses (Williams et al., 2015). Our observations of the phenotypic responses of alien plant species to urbanization are in line with interspecific trait variations for alien plants observed before, demonstrating that plant height is mostly influenced by land cover, while SLA depends on climate (Milanović et al., 2020). We further acknowledge that the phenotypic responses of the alien plants in our study can be either linked to trait plasticity or genetic diversity (Gorton et al., 2018; Rivkin et al., 2019) and probably result from the combination of both. We did not include germination or reproduction traits in our study, and however, contrasted responses for these traits could be detected for alien plants from various origin along the urban‐to‐rural gradients (Gorton et al., 2018). Nevertheless, significant future work will be needed to expand our understanding of intra‐ and interspecific trait variation along urban‐to‐rural gradients. Potential future avenues include studies on specific phenological stages such as germination or flowering, comparisons with native conspecifics, or even common garden experiments that allow for controlling certain environmental variables (such as soil type and/or soil moisture).

Our results highlight the need to study environmental conditions as close to the individual as possible, as those drivers were found to be important for alien plant growth and leaf variables, and not the coarser urban‐to‐rural gradient or climate. This provides insights for further studies trying to disentangle the effects of urban‐to‐rural gradients on alien plant's phenotypic responses (Godefroid & Ricotta, 2018; Williams et al., 2015). To do so, we emphasize the relevance of using various traits allowing to grasp both seasonal growth and shorter time steps such as warm summer periods. While native plant communities are forecasted to be further impacted by urbanization, climate change, and increasing recurrence of extreme events such as droughts or longer heat waves, plant invasions in urban areas are predicted to become more prevalent due to the lower biotic resistance of the city flora and sustained arrival of alien plant propagules by human activities (Catford et al., 2009; Gaertner et al., 2017; van Kleunen et al., 2018). A better understanding of alien plant responses along urban‐to‐rural gradients is therefore necessary, as even in the most urbanized areas and under warm and dry periods, shielded situations might provide shelter to alien plant species.

## CONFLICT OF INTEREST

The authors declare that the research was conducted in the absence of any commercial or financial relationships that could be construed as a potential conflict of interest.

## AUTHOR CONTRIBUTION

**Charly Géron:** Conceptualization (equal); Data curation (lead); Formal analysis (lead); Funding acquisition (lead); Investigation (lead); Methodology (equal); Project administration (lead); Resources (equal); Software (lead); Supervision (equal); Validation (lead); Visualization (lead); Writing‐original draft (lead); Writing‐review & editing (lead). **Jonas Lembrechts:** Conceptualization (equal); Data curation (supporting); Formal analysis (supporting); Funding acquisition (supporting); Investigation (supporting); Methodology (equal); Project administration (supporting); Resources (equal); Software (supporting); Supervision (equal); Validation (supporting); Visualization (supporting); Writing‐original draft (supporting); Writing‐review & editing (supporting). **Ivan Nijs:** Conceptualization (equal); Data curation (supporting); Formal analysis (supporting); Funding acquisition (supporting); Investigation (supporting); Methodology (equal); Project administration (supporting); Resources (equal); Software (supporting); Supervision (equal); Validation (supporting); Visualization (supporting); Writing‐original draft (supporting); Writing‐review & editing (supporting). **Arnaud Monty:** Conceptualization (equal); Data curation (supporting); Formal analysis (supporting); Funding acquisition (supporting); Investigation (supporting); Methodology (equal); Project administration (supporting); Resources (equal); Software (supporting); Supervision (equal); Validation (supporting); Visualization (supporting); Writing‐original draft (supporting); Writing‐review & editing (supporting).

## DATA AVAILABILITY STATEMENT

Preprocessed data made available on Figshare: https://doi.org/10.6084/m9.figshare.14816349.v1.

## Supporting information

Supplementary Material
