# Supplementary material for: Shielded environments reduce stress in alien Asteraceae species during hot and dry summers along urban‐to‐rural gradients
Source: Ecol Evol. 2021 Jul 13;11(15):10613–26. doi: 10.1002/ece3.7872 (PMC8328448; doi:10.1002/ece3.7872)
Supplement: Supplementary file 1 — Supplementary Material [file ECE3-11-10613-s001.docx]

**Appendices**

**Appendix 1:** The study area (corresponding to the Atlantic biogeographical region of Belgium (European Environment Agency (EEA) 2011) was situated north of the Meuse river. This spatial delineation was chosen in order to consider a study region with background climatic conditions as homogeneous as possible. Its extent is represented by the red rectangle of coordinates: top left 51°18’29’’N 3°7’26’’E, bottom right 50°30’58’’N 5°22’12’’E (WGS 84 coordinate system), and encompassed Flanders, Brussels Capital Region, and the north of Wallonia (Appendix 1). The locations of the studied organisms for the 2018 field season (red circles), and 2019 field season (yellow circles) are represented. From top to bottom: National Geographic Institute (NGI) map of Belgium, urbanity at the landscape scale (400 m²), and urbanity at the local scale (9 km²).


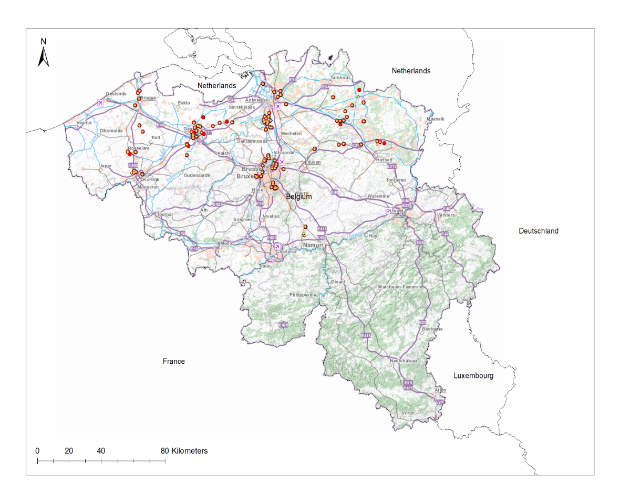

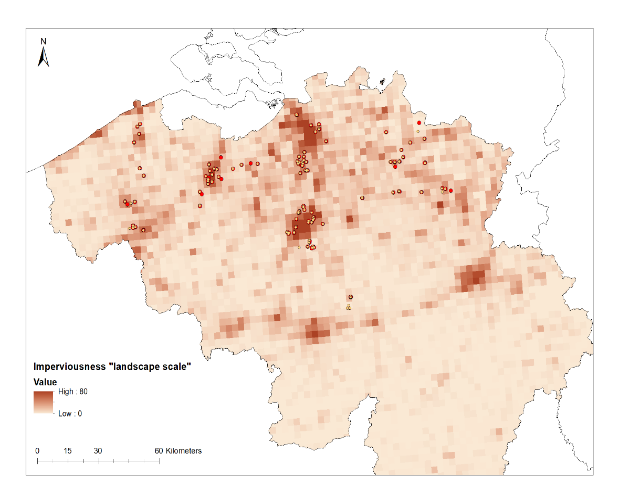

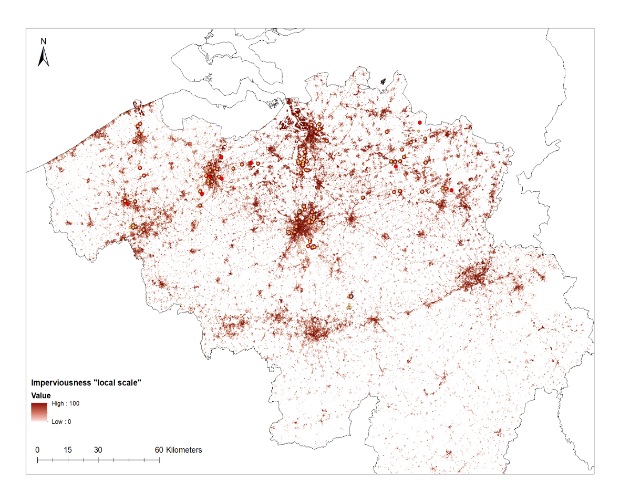


**Appendix 2:** Species distribution model detailed method

Native ranges of the six studied alien plant species were modelled to find their mean native climate preferences with a species distribution model framework.

Countries and regions within the native range for each of the six species were retrieved from Plants of the World Online (POWO 2020) and occurrences were acquired from the Global Biodiversity Information Facility (GBIF 2020) using the R packages rgbif and taxize (Chamberlain et al. 2019, 2020). Native regions were reported following the World Geographical Scheme for Recording Plant Distributions (WGSRPD) (Brummitt et al. 2001). In order to minimize spatial autocorrelation, the variability in number of occurrence points across different reporting countries was minimized by taking a maximum of 300 occurrence points retrieved from GBIF (GBIF Secretariat 2019) for each species per country within their respective native range. By default, this included only the most recent occurrence points. Then, only the points falling inside reported native regions were kept. Duplicates were removed, as well as outliers that were detected based on Mahalanobis distance (MD) and defined as any points having an MD four times larger than the 0.95 percentile of the entirety (Mahalanobis 1936). MD was calculated for each occurrence based on its location and bioclimatic conditions. Since outliers already influence the calculations of MD, we applied this approach again after removing the first set of outliers. Since species distributions hardly follow any administrative boundaries, we define the modelling extent for each studied alien plant species as all ecoregions (Olson et al. 2001) containing at least one of its remaining occurrence points plus all neighboring ecoregions, to acknowledge the fact that defining native countries and regions as well as data availability within ecoregions may be subject to errors and biases.

WorldClim bioclimatic predictors and land cover types were used to predict the possible native range of each species at 30 arcmin resolution across the defined modelling extent using maximum entropy modelling (Maxent) (Phillips et al. 2004, 2006, 2017). WorldClim bioclimatic predictors (n = 19, see https://www.worldclim.com/bioclim for full list) (Fick & Hijmans 2017) were downloaded in 10 arcmin resolution and aggregated to modelling resolution (30 arcmin, approximately 56 km at the equator) by averaging. Fractions of land cover classes (n = 22, including different types and mosaics of vegetation, see http://maps.elie.ucl.ac.be/CCI/viewer/download/CCI-LC_Maps_Legend.pdf for full list) were calculated based on the European Space Agency’s land cover product (original scale 300 m X 300 m, ESA 2017). Each class was transformed into a binary raster depicting presence (= 1) or absence (= 0) of the land cover type, and then aggregated to modelling resolution by averaging, resulting in one raster for each land cover class. Handling of spatial data was conducted using the R packages raster, rgdal, maptools, rgeos and sp (Bivand et al. 2013, 2019; Bivand & Lewin-Koh 2019; Bivand & Rundel 2019; Hijmans 2019).

Maxent is a probability density estimation approach suited for predicting species distributions based on presence-only data (Elith et al. 2006). In Maxent, environmental variables and transformations thereof are used to predict species distributions. For our native range predictions, we kept the Maxent default settings for selecting appropriate variables, transformations (out of linear, quadratic, product, hinge and threshold) and regularization values used to reduce overfitting (Elith et al. 2011; Merow et al. 2013). In addition, Maxent requires background information to contrast the environmental background against presence locations, and ultimately, fit response curves (Elith et al. 2011). We collected this background information (WorldClim bioclimatic predictors and land cover classes) from each cell across the modelling extent to avoid fitting the model with randomly generated points. Model fitting, prediction and range generation were executed with the R package dismo (Hijmans et al. 2017). The Maxent native range prediction was transformed into presence cells using the cut-off threshold that maximized model sensitivity for each species. This step resulted in a patchy landscape consisting of suitable and unsuitable habitat. We then selected all suitable habitat patches that contained or were in the proximity (i.e. a distance from the closest occurrence point of up to 10% of the total extent of the suitable patch) of at least one occurrence point, hence, removing patches that are unlikely being colonized by the species. This gave us a coarse native range area estimation for each of the studied alien plant species.

**Appendix 3:** Maxent native range prediction models information for the six studied species

| Species | *Artemisia verlotiorum* | *Erigeron canadensis* | *Galinsoga quadriradiata* | *Matricaria discoidea* | *Senecio inaequidens* | *Solidago gigantea* |
| --- | --- | --- | --- | --- | --- | --- |
| Kappa | 0.91 | 0.99 | 0.96 | 0.99 | 0.96 | 1 |
| Species - sensitivity | 0.66 | 0.11 | 0.19 | 0.35 | 0.49 | 0.27 |
| No omission | 0.66 | 0.09 | 0.19 | 0.35 | 0.09 | 0.05 |
| Prevalence | 0.002 | 0.01 | 0.01 | <0.001 | 0.01 | 0.003 |
| Equal sensitivity and specificity | 0.66 | 0.15 | 0.27 | 0.36 | 0.31 | 0.22 |
| Sensitivity | 0.67 | 0.15 | 0.3 | 0.36 | 0.52 | 0.11 |
| AUC | 0.99 | 0.98 | 0.98 | 0.98 | 0.99 | 0.93 |
| Correlation | 0.18 | 0.41 | 0.49 | 0.09 | 0.52 | 0.20 |
| Maximum true positive rate + true negative rate | 0.66 | 0.16 | 0.19 | 0.36 | 0.50 | 0.27 |

**Appendix 4:** Principal component analysis (PCA) additional information

**
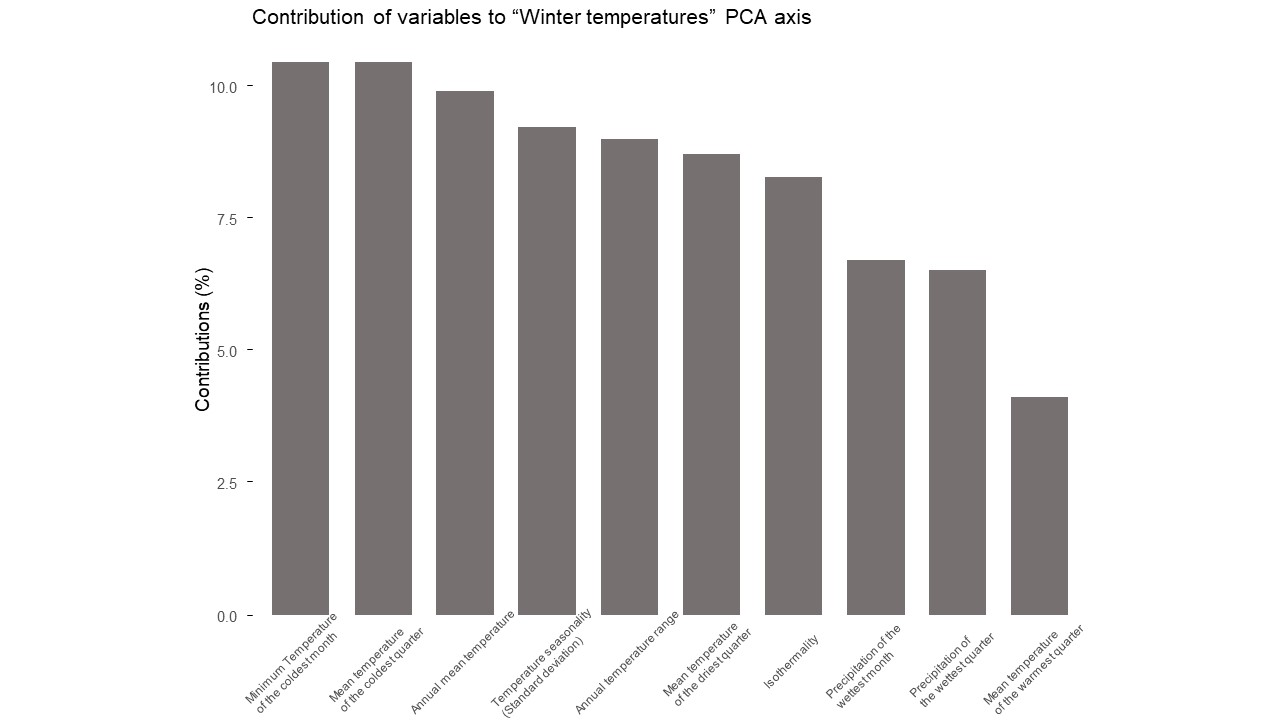
**

Figure 1: Histogram of the contributions of the bioclimatic predictors accounting to the first axis of the PCA of the native climatic conditions over the modelled native ranges of the studied alien plant species, called “winter temperatures” (See Methods for more details). The minimum temperature of the coldest month (Bio 6), the minimum temperature of the coldest quarter (Bio 11) and the annual mean temperature (Bio 1) were the three bioclimatic predictors contributing the most to the first PCA axis.

Table 1: Correlations of the bioclimatic predictors to the first axis of the “winter temperatures” PCA axis of the climatic conditions over the modelled native ranges of the studied alien plant species. Bioclimatic predictors are ordered from the highest to the lowest correlation to the “winter temperatures” axis. The three bioclimatic predictors contributed the most to the “winter temperatures” axis are in bold.

| Bioclimatic predictors | Correlation to "winter temperatures" PCA axis |
| --- | --- |
| **Minimum temperature of the coldest month** | **0.95** |
| **Mean temperature of the coldest quarter** | **0.93** |
| Isothermality | 0.85 |
| **Annual mean temperature** | **0.80** |
| Precipitation of the wettest month | 0.77 |
| Precipitation of the wettest quarter | 0.76 |
| Mean temperature of the driest quarter | 0.74 |
| Annual precipitation | 0.59 |
| Precipitation seasonality | 0.49 |
| Precipitation of the coldest quarter | 0.45 |
| Precipitation of the warmest quarter | 0.23 |
| Mean temperature of the warmest quarter | 0.13 |
| Mean temperature of the wettest quarter | 0.10 |
| Maximum of the warmest month | 0.02 |
| Precipitation of the driest quarter | <0.01 |
| Precipitation of the driest month | <0.01 |
| Annual mean diurnal range | -0.23 |
| Annual mean diurnal range | -0.93 |
| Temperature seasonality | -0.94 |

**Appendix 5:** Spearman correlations

Table 1: Spearman correlations between the urbanity at the landscape, local and organism scales as well as the vegetation cover and the SVF. Correlations statistically significant are in bold.

|  | SVF | Vegetation cover | Urbanity organism | Urbanity local |
| --- | --- | --- | --- | --- |
| Urbanity landscape | S = 19513297, p-value = 0.14, rho = 0.07 | **S = 24865870, p-value < 0.001, rho = - 0.20** | **S = 15928910, p-value < 0.001, rho = 0.16** | **S = 13540134, p-value < 0.001, rho = 0.30** |
| Urbanity local | S = 17720146, p-value = 0.48, rho = 0.03 | **S = 26357368, p-value < 0.001, rho = - 0.38** | **S = 13513114, p-value < 0.001, rho = 0.29** | / |
| Urbanity organism | S = 18680767, p-value = 0.48, rho = -0.03 | **S = 21127203, p-value = 0.01, rho = - 0.11** | / | / |
| Vegetation cover | S = 19513297, p-value = 0.12, rho = -0.07 | / | / | / |


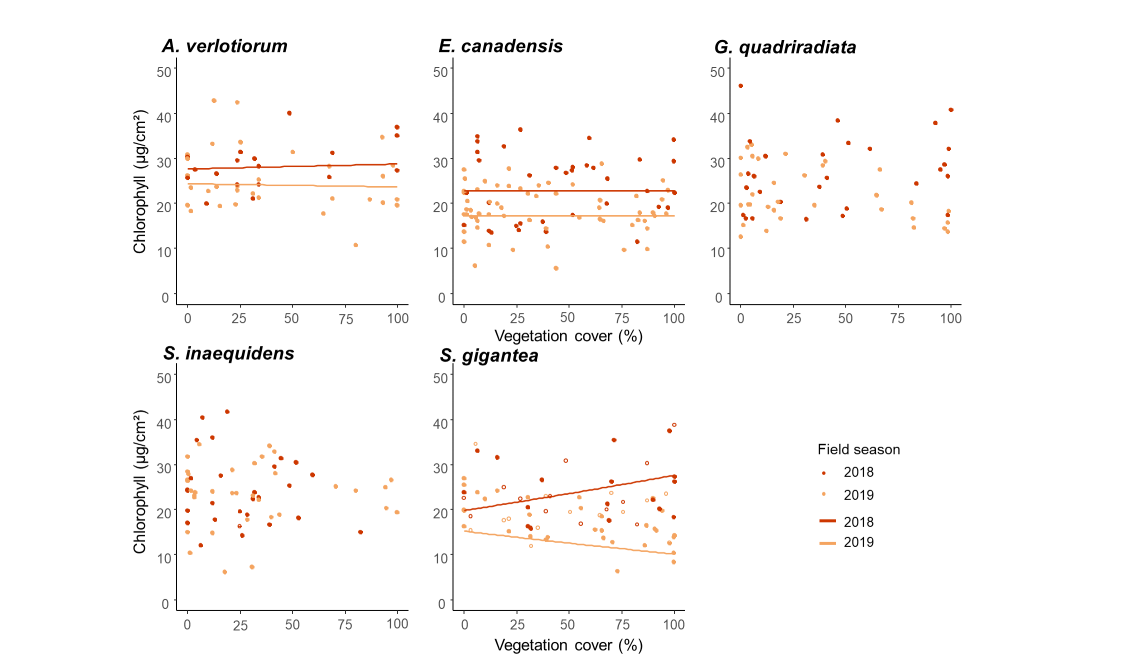
**Appendix 6:** Additional figures and tables

Figure 1: Chlorophyll content as a function of the vegetation cover, for the alien Asteraceae species*.* Measured values (dots) and modelled trends (lines) reported for 2018 (red) and 2019 (orange) separately, from the species-specific averaged models (See Appendix 6, Table 5). If the vegetation cover was not included in the species-specific averaged models, no trendlines were drawn.

Figure 2: Internode space as a function of urbanity at the “organism scale” (%, measured in a 40  40 cm square around the studied individual), for the alien Asteraceae species*.* Measured values (dots) and modelled trends (lines) reported for 2018 (red) and 2019 (orange) separately, from the species-specific averaged models (See Appendix 6, Table 6).

Table 1: Estimates, standard errors and Z values for the averaged models testing for the drivers of the specific leaf area (SLA) conducted for each species separately. Significant effects are in bold, and coded as follows: *** = p < 0.001; ** = p < 0.01; * = p < 0.05. N corresponds to the number of observations for each variable. Numbers in italic correspond to the variables or interactions present in the model with the lowest AIC, and significance codes between brackets correspond to significance in model with the lowest AIC. Blank cells correspond to explanatory variables or interactions not present in the averaged model. Urb. organism for the percentage of impervious surfaces (urbanity) at the organism scale (40 X 40 cm); Urb. local for the urbanity at the local scale (20 X 20 m); Urb. landscape for the urbanity at the landscape scale (3 X 3 km).

| **Specific leaf area (SLA)** | ***A. verlotiorum*** | ***E. canadensis*** | ***G. quadriradiata*** |  |  | ***M. discoidea*** | ***S. inaequidens*** |  | ***S. gigantea*** |
| --- | --- | --- | --- | --- | --- | --- | --- | --- | --- |
| (Intercept) | ***6.35^***(***)^*** | ***5.79^***(***)^*** | ***5.98^***(***)^*** |  |  | ***5.79^***(***)^*** | ***5.75^***(***)^*** |  | ***5.21^***(***)^*** |
|  | (0.72) | (0.62) | (0.83) |  |  | (0.62) | (0.22) |  | (0.21) |
| Urb. organism |  | *<-0.001^(*)^* |  |  |  | *<-0.001^(*)^* | <-0.001 |  | <0.001 |
|  |  | (<0.001) |  |  |  | (<0.001) | (<0.001) |  | (<0.001) |
| Urb. local | <-0.001 | *<0.001* |  |  |  | *<0.001* |  |  |  |
|  | (<0.001) | (<0.001) |  |  |  | (<0.001) |  |  |  |
| Urb. landscape | <0.001 | *0.01^(*)^* | *0.01* |  |  | *0.01^(**)^* |  |  | <0.001 |
|  | (<0.001) | (0.02) | (0.01) |  |  | (0.02) |  |  | (<0.001) |
| SVF | *-2.79(*)* | -1.01 | -0.88 |  |  | -1.01 | *-0.58^(**)^* |  | *-0.20* |
|  | (2.51) | (2.37) | (2.32) |  |  | (2.37) | (0.57) |  | (0.33) |
| SVF^2 | *1.63* | *0.74* | 0.28 |  |  | *0.74* | -0.42 |  | -0.11 |
|  | (2.09) | (2.27) | (1.93) |  |  | (2.27) | (0.51) |  | (0.22) |
| Vegetation cover | -0.10 | 0.12 | 0.25 |  |  | 0.12 | -0.03 |  | 0.04 |
|  | (0.25) | (0.59) | (0.51) |  |  | (0.59) | (0.10) |  | (0.26) |
| Field season 2019 | ***-1.01^***(***)^*** | *-1.19^(***)^* | ***-1.42^**(***)^*** |  |  | *-1.19^(***)^* | ***-0.98^***(***)^*** |  | ***-0.88^***(***)^*** |
|  | (0.08) | (0.98) | (0.51) |  |  | (0.98) | (0.16) |  | (0.15) |
| Urb. landscape 🞫 Urb. organism |  | <0.001 |  |  |  | <0.001 |  |  | <-0.001 |
|  |  | (<0.001) |  |  |  | (<0.001) |  |  | (<0.001) |
| Urb. local 🞫 Urb. landscape | <-0.001 | *<-0.001* |  |  |  | *<-0.001* |  |  |  |
|  | (<0.001) | (<0.001) |  |  |  | (<0.001) |  |  |  |
| Urb. organism 🞫 SVF |  | <0.001 |  |  |  | <0.001 |  |  |  |
|  |  | (<0.001) |  |  |  | (<0.001) |  |  |  |
| Urb. local 🞫 SVF | <-0.001 |  |  |  |  |  |  |  |  |
|  | (<0.001) |  |  |  |  |  |  |  |  |
| Urb. landscape 🞫 SVF |  | <0.001 |  |  |  | <0.001 |  |  |  |
|  |  | (0.05) |  |  |  | (0.05) |  |  |  |
| SVF^2 🞫 Urb. local | <-0.001 |  |  |  |  |  |  |  |  |
|  | (<0.001) |  |  |  |  |  |  |  |  |
| SVF^2 🞫 Urb. landscape |  | *-0.03^(*)^* |  |  |  | ***-0.03**** |  |  |  |
|  |  | (0.05) |  |  |  | (0.05) |  |  |  |
| Urb. local 🞫 Field season 2019 | <0.001 | <-0.001 |  |  |  | <-0.001 |  |  |  |
|  | (<0.001) | (<0.001) |  |  |  | (<0.001) |  |  |  |
| Urb. landscape 🞫 Field season 2019 |  | <-0.001 | <0.001 |  |  | <-0.001 |  |  |  |
|  |  | (<0.001) | (0.01) |  |  | (<0.001) |  |  |  |
| SVF 🞫 Field season 2019 |  | 1.97 |  |  |  | 1.97 | -0.03 |  |  |
|  |  | (3.68) |  |  |  | (3.68) | (0.23) |  |  |
| SVF^2 🞫 Field season 2019 |  | -2.03 |  |  |  | -2.03 |  |  |  |
|  |  | (3.34) |  |  |  | (3.34) |  |  |  |
| Urb. landscape 🞫 Vegetation cover |  |  | <-0.001 |  |  |  |  |  |  |
|  |  |  | (0.01) |  |  |  |  |  |  |
| SVF 🞫 Vegetation cover | 0.07 | -0.43 |  |  |  | -0.43 |  |  | -0.06 |
|  | (0.34) | (2.35) |  |  |  | (2.35) |  |  | (0.35) |
| SVF^2 🞫 Vegetation cover | 0.07 | 0.45 |  |  |  | 0.45 |  |  |  |
|  | (0.32) | (2.27) |  |  |  | (2.27) |  |  |  |
| Vegetation cover 🞫 Field season 2019 |  | <0.001 | *0.75* |  |  | <0.001 |  |  | ***0.56^*(*)^*** |
|  |  | (0.04) | (0.77) |  |  | (0.04) |  |  | (0.24) |
| Model with the lowest AIC information | | | | | | | | | |
| R² | 0.84 | 0.69 | 0.36 |  |  | 0.69 | 0.63 |  | 0.46 |
| Num. obs. | 53 | 107 | 70 |  |  | 68 | 72 |  | 86 |
| ^***^p < <0.0011; ^**^p < 0.01; ^*^p < 0.05 | | | | | | | | | |
|  |  |  |  |  | | | | | |

Table 2: Estimates, standard errors and Z values for the averaged models testing for the drivers of the anthocyanins index conducted for each species separately. Significant effects are in bold, and coded as follows: *** = p < 0.001; ** = p < 0.01; * = p < 0.05. N corresponds to the number of observations for each variable. Numbers in italic correspond to the variables or interactions present in the model with the lowest AIC, and significance codes between brackets correspond to significance in the model with the lowest AIC. Blank cells correspond to explanatory variables or interactions not present in the averaged model. Urb. organism for the percentage of impervious surfaces (urbanity) at the organism scale (40 X 40 cm); Urb. local for the urbanity at the local scale (20 X 20 m); Urb. landscape for the urbanity at the landscape scale (3 X 3 km).

|  |  |  |  |  | **Anthocyanins** | ***A. verlotiorum*** | ***E. canadensis*** | ***G. quadriradiata*** | ***S. inaequidens*** | ***S. gigantea*** | |
| --- | --- | --- | --- | --- | --- | --- | --- | --- | --- | --- | --- |
|  |  |  |  |  | (Intercept) | ***0.13^*(***)^*** | *0.14* | *0.07* | ***0.40^***(***)^*** | *0.15* | |
|  |  |  |  |  |  | (0.06) | (0.14) | (0.07) | (0.10) | (0.11) | |
|  |  |  |  |  | Urb. organism | *<0.001^(***)^* |  | ***<0.001^*(*)^*** | *<0.001* | *<-0.001* | |
|  |  |  |  |  |  | (0.01) |  | (<0.001) | (<0.001) | (<0.001) | |
|  |  |  |  |  | Urb. local | *<0.001* |  |  | ***<-0.001^**(**)^*** |  | |
|  |  |  |  |  |  | (<0.001) |  |  | (<0.001) |  | |
|  |  |  |  |  | Urb. landscape |  | *<0.001^(*)^* |  | ***-0.01^***(***)^*** |  | |
|  |  |  |  |  |  |  | (<0.001) |  | (<0.001) |  | |
|  |  |  |  |  | SVF | *0.05* | *0.11^(*)^* | *0.04* |  | *0.16^(**)^* | |
|  |  |  |  |  |  | (0.09) | (0.19) | (0.08) |  | (0.21) | |
|  |  |  |  |  | SVF^2 | 0.04 | 0.07 | 0.03 |  | 0.14 | |
|  |  |  |  |  |  | (0.08) | (0.15) | (0.07) |  | (0.16) | |
|  |  |  |  |  | Vegetation cover | *-0.05* | -0.05 | *0.08* |  | *0.01* | |
|  |  |  |  |  |  | (0.05) | (0.08) | (0.09) |  | (0.16) | |
|  |  |  |  |  | Field season 2019 | *<-0.001* | *0.06^(***)^* | ***0.09^**(**)^*** | *-0.06* | ***0.11^**(***)^*** | |
|  |  |  |  |  |  | (0.02) | (0.07) | (0.03) | (0.07) | (0.04) | |
|  |  |  |  |  | Urb. organism 🞫 Urb. local | *<-0.001^(**)^* |  |  | *<0.001* |  | |
|  |  |  |  |  |  | (<0.001) |  |  | (<0.001) |  | |
|  |  |  |  |  | Urb. local 🞫 Urb. landscape |  |  |  | ***<0.001^**(**)^*** |  | |
|  |  |  |  |  |  |  |  |  | (<0.001) |  | |
|  |  |  |  |  | Urb. organism 🞫 SVF | <0.001 |  |  |  |  | |
|  |  |  |  |  |  | (0.01) |  |  |  |  | |
|  |  |  |  |  | Urb. landscape 🞫 SVF |  | *<-0.001* |  |  |  | |
|  |  |  |  |  |  |  | (<0.001) |  |  |  | |
|  |  |  |  |  | Urb. organism 🞫 SVF^2 | <0.001 |  |  |  |  | |
|  |  |  |  |  |  | (<0.001) |  |  |  |  | |
|  |  |  |  |  | Urb. landscape 🞫 SVF^2 |  | <-0.001 |  |  |  | |
|  |  |  |  |  |  |  | (<0.001) |  |  |  | |
|  |  |  |  |  | Urb. organism 🞫 Field season 2019 | *<-0.001^(*)^* |  |  |  | *<-0.001* | |
|  |  |  |  |  |  | (<0.001) |  |  |  | (<0.001) | |
|  |  |  |  |  | Urb. landscape 🞫 Field season 2019 |  | <-0.001 |  | ***0.01^*(*)^*** |  | |
|  |  |  |  |  |  |  | (<0.001) |  | (<0.001) |  | |
|  |  |  |  |  | SVF 🞫 Field season 2019 |  | 0.02 |  |  |  | |
|  |  |  |  |  |  |  | (0.08) |  |  |  | |
|  |  |  |  |  | SVF^2 🞫 Field season 2019 |  | 0.01 |  |  |  | |
|  |  |  |  |  |  |  | (0.06) |  |  |  | |
|  |  |  |  |  | Urb. organism 🞫 Vegetation cover | *<-0.001* |  | ***<-0.001^*(*)^*** |  | *<0.001^(*)^* | |
|  |  |  |  |  |  | (<0.001) |  | (<0.001) |  | (<0.001) | |
|  |  |  |  |  | Urb. local 🞫 Vegetation cover | <-0.001 |  |  |  |  | |
|  |  |  |  |  |  | (<0.001) |  |  |  |  | |
|  |  |  |  |  | SVF 🞫Vegetation cover |  | -0.01 |  |  | -0.13 | |
|  |  |  |  |  |  |  | (0.07) |  |  | (0.27) | |
|  |  |  |  |  | SVF^2 🞫 Vegetation cover |  |  |  |  | *-0.08* | |
|  |  |  |  |  |  |  |  |  |  | (0.20) | |
|  |  |  |  |  | Vegetation cover 🞫 Field season 2019 | 0.01 | 0.07 |  |  | 0.01 | |
|  |  |  |  |  |  | (0.03) | (0.09) |  |  | (0.03) | |
|  |  |  |  | Model with the lowest AIC information | | | | | | |  |
|  |  |  |  |  | R² | 0.34 | 0.23 | 0.28 | 0.36 | 0.35 | |
|  |  |  |  |  | Num. obs. | 51 | 96 | 59 | 62 | 77 | |
|  |  |  |  |  | ^***^p < <0.0011; ^**^p < 0.01; ^*^p < 0.05 | | | | | | |

Table 3: Estimates, standard errors and Z values for the averaged models testing for the drivers of the flavonols index conducted for each species separately. Significant effects are in bold, and coded as follows: *** = p < 0.001; ** = p < 0.01; * = p < 0.05. N corresponds to the number of observations for each variable. Numbers in italic correspond to the variables or interactions present in the model with the lowest AIC, and significance codes between brackets correspond to significance in the model with the lowest AIC. Blank cells correspond to explanatory variables or interactions not present in the averaged model. Urb. organism for the percentage of impervious surfaces (urbanity) at the organism scale (40 X 40 cm); Urb. local for the urbanity at the local scale (20 X 20 m); Urb. landscape for the urbanity at the landscape scale (3 X 3 km).

| **Flavonols** | ***A. verlotiorum*** | ***E. canadensis*** | ***G. quadriradiata*** | ***S. inaequidens*** | ***S. gigantea*** |
| --- | --- | --- | --- | --- | --- |
| (Intercept) | *0.30* | ***0.28^*^*** | *-0.39* | *0.21* | ***0.82^***(***)^*** |
|  | (0.19) | (0.11) | (0.76) | (0.15) | (0.08) |
| Urb. organism | <0.001 | <0.001 | *<-0.001* |  | ***<-0.001^*(*)^*** |
|  | (<0.001) | (<0.001) | (0.01) |  | (<0.001) |
| Urb. local | *<0.001* |  | ***-0.01^*(*)^*** |  | *<0.001* |
|  | (<0.001) |  | (<0.001) |  | (<0.001) |
| Urb. landscape | ***<-0.001^**(***)^*** |  |  |  |  |
|  | (<0.001) |  |  |  |  |
| SVF | *0.63^(***)^* | *0.34^(***)^* | *2.02^(*)^* | *0.31^(**)^* | -0.01 |
|  | (0.41) | (0.34) | (2.76) | (0.35) | (0.10) |
| SVF^2 | 0.13 | 0.27 | *-1.09* | 0.28 | *0.01* |
|  | (0.27) | (0.29) | (2.33) | (0.32) | (0.07) |
| Vegetation cover | *-0.25^(*)^* | -0.01 | ***-1.04^*(**)^*** | -0.02 | *-0.01* |
|  | (0.16) | (0.03) | (0.49) | (0.06) | (0.09) |
| Field season 2019 | *0.21* | ***-0.21^***(***)^*** |  | ***-0.50^**(***)^*** | ***-0.16^***(***)^*** |
|  | (0.18) | (0.03) |  | (0.19) | (0.03) |
| Urb. organism 🞫 Urb. local |  |  | ***<0.001^**(**)^*** |  |  |
|  |  |  | (<0.001) |  |  |
| Urb. organism 🞫 SVF |  |  | 0.01 |  | <0.001 |
|  |  |  | (0.03) |  | (<0.001) |
| Urb. local 🞫 SVF |  |  | <-0.001 |  |  |
|  |  |  | (<0.001) |  |  |
| Urb. organism 🞫 SVF^2 |  |  | -0.01 |  | *<0.001^(*)^* |
|  |  |  | (0.03) |  | (<0.001) |
| Urb. local 🞫 SVF^2 |  |  | <-0.001 |  |  |
|  |  |  | (<0.001) |  |  |
| SVF 🞫 Field season 2019 | *-0.41^(*)^* |  |  | 0.10 |  |
|  | (0.32) |  |  | (0.28) |  |
| SVF^2 🞫 Field season 2019 | -0.08 |  |  | 0.10 |  |
|  | (0.19) |  |  | (0.26) |  |
| Urb. local 🞫 Field season 2019 | <0.001 |  |  |  | *<-0.001* |
|  | (<0.001) |  |  |  | (<0.001) |
| Urb. organism 🞫 Field season 2019 |  |  |  |  | <-0.001 |
|  |  |  |  |  | (<0.001) |
| SVF 🞫 Vegetation cover | 0.07 |  | *1.80^(**)^* |  | 0.04 |
|  | (0.23) |  | (1.56) |  | (0.13) |
| SVF^2 🞫 Vegetation cover | 0.07 |  | 0.13 |  | 0.01 |
|  | (0.22) |  | (1.42) |  | (0.07) |
| Urb. local 🞫 Vegetation cover |  |  |  |  | ***<-0.001^*(*)^*** |
|  |  |  |  |  | (<0.001) |
| Vegetation cover 🞫 Field season 2019 | *0.15^(*)^* |  |  |  |  |
|  | (0.13) |  |  |  |  |
| Model with the lowest AIC information | | | | | |
| R² | 0.58 | 0.40 | 0.69 | 0.50 | 0.65 |
| Num. obs. | 51 | 96 | 61 | 62 | 77 |
| ^***^p < <0.0011; ^**^p < 0.01; ^*^p < 0.05 | | | | | |

Table 4: Estimates, standard errors and Z values for the averaged models testing for the drivers of plant height conducted for each species separately. Significant effects are in bold, and coded as follows: *** = p < 0.001; ** = p < 0.01; * = p < 0.05. N corresponds to the number of observations for each variable. Numbers in italic correspond to the variables or interactions present in the model with the lowest AIC, and significance codes between brackets correspond to significance in the model with the lowest AIC. Blank cells correspond to explanatory variables or interactions not present in the averaged model. Urb. organism for the percentage of impervious surfaces (urbanity) at the organism scale (40 X 40 cm); Urb. local for the urbanity at the local scale (20 X 20 m); Urb. landscape for the urbanity at the landscape scale (3 X 3 km).

| **Height** | ***A. verlotiorum*** | ***E. canadensis*** | ***G. quadriradiata*** | ***M. discoidea*** | ***S. inaequidens*** | ***S. gigantea*** |
| --- | --- | --- | --- | --- | --- | --- |
| (Intercept) | *3.61^(***)^* | ***5.17^***(***)^*** | ***2.87^***(***)^*** | ***2.52^***(***)^*** | ***4.43^***(***)^*** | ***4.59^***(***)^*** |
|  | (1.89) | (0.84) | (0.36) | (0.18) | (0.49) | (0.22) |
| Urb. organism | ***-0.74^***(***)^*** | *<-0.001^(*)^* | *<0.001* | *<-0.001^(*)^* | ***-0.01^**(**)^*** | *<-0.001* |
|  | (0.19) | (0.01) | (<0.001) | (<0.001) | (<0.001) | (<0.001) |
| Urb. local | *<-0.001* | <0.001 |  | <-0.001^(*)^ |  | <-0.001 |
|  | (0.01) | (<0.001) |  | (<0.001) |  | (<0.001) |
| Urb. landscape |  | *-0.03* | *<0.001* |  | <-0.001 |  |
|  |  | (0.03) | (0.01) |  | (<0.001) |  |
| SVF | *4.27* | *-2.17^(*)^* | 0.05 | -0.03 | -0.41 | 0.03 |
|  | (6.89) | (1.64) | (0.29) | (0.22) | (1.68) | (0.17) |
| SVF^2 | *-3.92* | -0.03 | 0.03 | -0.03 | 0.30 | 0.03 |
|  | (6.02) | (0.17) | (0.23) | (0.12) | (1.40) | (0.15) |
| Vegetation cover | 1.91 | *0.62* | 0.07 |  | -0.06 | *0.26* |
|  | (3.03) | (0.53) | (0.56) |  | (0.17) | (0.26) |
| Field season 2019 | 0.22 | ***-1.10^**(***)^*** | *-0.87^(***)^* |  | -0.68 | *-0.15* |
|  | (0.44) | (0.33) | (0.46) |  | (1.91) | (0.14) |
| Urb. organism 🞫 Urb. landscape |  |  | <0.001 |  |  |  |
|  |  |  | (<0.001) |  |  |  |
| Urb. organism 🞫 Urb. local | ***<0.001^**(***)^*** |  |  | <-0.001 |  |  |
|  | (<0.001) |  |  | (<0.001) |  |  |
| Urb. organism 🞫 SVF | ***2.18^***(***)^*** | <-0.001 |  |  |  |  |
|  | (0.59) | (0.01) |  |  |  |  |
| Urb. local 🞫 SVF | <0.001 |  |  | <-0.001 |  |  |
|  | (0.01) |  |  | (0.01) |  |  |
| Urb. landscape 🞫 SVF |  | *0.06^(*)^* |  |  |  |  |
|  |  | (0.05) |  |  |  |  |
| Urb. organism 🞫 SVF^2 | ***-1.65^***(***)^*** |  |  |  |  |  |
|  | (0.44) |  |  |  |  |  |
| Urb. organism 🞫 Field season 2019 |  | *<0.001* | *<0.001* |  |  |  |
|  |  | (<0.001) | (0.01) |  |  |  |
| Urb. local 🞫 Field season 2019 |  |  |  | 0.01^(*)^ |  |  |
|  |  |  |  | (<0.001) |  |  |
| SVF 🞫 Field season 2019 | -0.35 |  |  |  | 2.20 |  |
|  | (0.71) |  |  |  | (6.74) |  |
| SVF^2 🞫 Field season 2019 |  |  |  |  | -1.87 |  |
|  |  |  |  |  | (5.77) |  |
| Urb. organism 🞫 Vegetation cover | <0.001 |  |  |  |  | ***-0.01^**(**)^*** |
|  | (0.01) |  |  |  |  | (<0.001) |
| Urb. local 🞫 Vegetation cover |  |  |  |  |  | <-0.001 |
|  |  |  |  |  |  | (<0.001) |
| Urb. landscape 🞫 Vegetation cover |  | *-0.03^(*)^* |  |  |  |  |
|  |  | (0.02) |  |  |  |  |
| SVF 🞫 Vegetation cover | -7.91 |  | -0.13 |  |  |  |
|  | (11.54) |  | (0.71) |  |  |  |
| SVF^2 🞫 Vegetation cover | 7.55 |  | -0.11 |  |  |  |
|  | (10.39) |  | (0.63) |  |  |  |
| Vegetation cover 🞫 Field season 2019 |  | 0.04 | -0.27 |  |  |  |
|  |  | (0.19) | (0.48) |  |  |  |
| R² | 0.66 | 0.32 | 0.29 | 0.21 | 0.14 | 0.42 |
| Num. obs. | 54 | 112 | 72 | 68 | 74 | 93 |
|  | ^***^p < <0.0011; ^**^p < 0.01; ^*^p < 0.05 | | | | | |

Table 5: Estimates, standard errors and Z values for the averaged models testing for the drivers of the chlorophyll content conducted for each species separately, as well as the residual analysis conducted on the best model for each species. Significant effects are in bold, and coded as follows: *** = p < 0.001; ** = p < 0.01; * = p < 0.05. N corresponds to the number of observations for each variable. Numbers in italic correspond to the variables or interactions present in the model with the lowest AIC, and significance codes between brackets correspond to significance in the model with the lowest AIC. Blank cells correspond to explanatory variables or interactions not present in the averaged model. Urb. organism for the percentage of impervious surfaces (urbanity) at the organism scale (40 X 40 cm); Urb. local for the urbanity at the local scale (20 X 20 m); Urb. landscape for the urbanity at the landscape scale (3 X 3 km).

| **Chlorophyll content** | ***A. verlotiorum*** | ***E. canadensis*** | ***G. quadriradiata*** | ***S. inaequidens*** | ***S. gigantea*** |
| --- | --- | --- | --- | --- | --- |
| (Intercept) | ***5.29^***(***)^*** | ***6.49^***(***)^*** | ***4.16^***(***°^*** | ***4.67^***(***)^*** | ***3.81^***(***)^*** |
|  | (0.34) | (0.90) | (0.65) | (0.23) | (0.60) |
| Urb. organism |  | <0.001 | *0.01* |  | *<0.001* |
|  |  | (<0.001) | (0.01) |  | (0.01) |
| Urb. local | *<-0.001* | <0.001 | <0.001 | <0.001 | *0.01^(*)^* |
|  | (<0.001) | (<0.001) | (<0.001) | (<0.001) | (0.01) |
| Urb. landscape |  | ***-0.04^*(**)^*** | <-0.001 | <0.001 | *0.01* |
|  |  | (0.02) | (<0.001) | (<0.001) | (0.01) |
| SVF | 0.08 | -1.82 | 2.32^(***)^ | 0.06 | *0.01* |
|  | (0.40) | (2.76) | (1.88) | (0.28) | (0.71) |
| SVF^2 | 0.08 | *-1.09^(**)^* | 1.16 | 0.06 | -0.24 |
|  | (0.37) | (2.49) | (1.62) | (0.27) | (0.60) |
| Vegetation cover | 0.10 | -0.01 |  |  | *0.94^(*)^* |
|  | (0.31) | (0.07) |  |  | (0.71) |
| Field season 2019 | *-0.14^(*)^* | ***-0.61^**(***)^*** | ***-1.60^***(***)^*** |  | *0.72^(*)^* |
|  | (0.58) | (0.23) | (0.34) |  | (0.42) |
| Urb. organism 🞫 Urb. landscape |  |  |  |  | *<0.001^(*)^* |
|  |  |  |  |  | (<0.001) |
| Urb. local 🞫 Urb. landscape |  |  |  |  | <-0.001 |
|  |  |  |  |  | (<0.001) |
| Urb. organism 🞫 SVF |  |  | *-0.02^(**)^* |  | *-0.01^(*)^* |
|  |  |  | (0.02) |  | (0.02) |
| Urb. organism 🞫 SVF^2 |  |  | -0.01 |  |  |
|  |  |  | (0.02) |  |  |
| Urb. organism 🞫 Field season 2019 |  |  | ***0.02^***(***)^*** |  | <0.001 |
|  |  |  | (<0.001) |  | (<0.001) |
| Urb. local 🞫 Field season 2019 | *0.01^(*)^* |  |  |  |  |
|  | (0.01) |  |  |  |  |
| Urb. landscape 🞫 SVF |  | 0.03 |  |  |  |
|  |  | (0.05) |  |  |  |
| SVF^2 🞫 Urb. landscape |  | *0.05^(*)^* |  |  |  |
|  |  | (0.05) |  |  |  |
| Urb. landscape 🞫 Field season 2019 |  | <-0.001 |  |  | ***-0.02^**(**)^*** |
|  |  | (<0.001) |  |  | (0.01) |
| SVF 🞫 Field season 2019 | -0.24 |  |  |  |  |
|  | (0.84) |  |  |  |  |
| SVF^2 🞫 Field season 2019 | -0.26 | 0.06 |  |  |  |
|  | (0.80) | (0.33) |  |  |  |
| Urb. local 🞫 Vegetation cover |  |  |  |  | *-0.01^(*)^* |
|  |  |  |  |  | (0.01) |
| Urb. landscape 🞫 Vegetation cover |  |  |  |  | *0.01^(*)^* |
|  |  |  |  |  | (0.01) |
| SVF 🞫 Vegetation cover |  |  |  |  | 0.13 |
|  |  |  |  |  | (0.66) |
| SVF^2 🞫 Vegetation cover |  |  |  |  | 0.24 |
|  |  |  |  |  | (0.83) |
| Vegetation cover 🞫 Field season 2019 | -0.18 |  |  |  | ***-1.53^**(***)^*** |
|  | (0.49) |  |  |  | (0.46) |
| Model with the lowest AIC information | | | | | |
| R² | 0.14 | 0.21 | 0.42 | 0 | 0.52 |
| Num. obs. | 51 | 96 | 61 | 62 | 77 |
| ^***^p < <0.0011; ^**^p < 0.01; ^*^p < 0.05 | | | | | |

Table 6: Estimates, standard errors and Z values for the averaged models testing for the drivers of the internode space conducted for each species separately, as well as the residual analysis conducted on the best model for each species. Significant effects are in bold, and coded as follows: *** = p < 0.001; ** = p < 0.01; * = p < 0.05. N corresponds to the number of observations for each variable. Numbers in italic correspond to the variables or interactions present in the model with the lowest AIC, and significance codes between brackets correspond to significance in the model with the lowest AIC. Blank cells correspond to explanatory variables or interactions not present in the averaged model. Urb. organism for the percentage of impervious surfaces (urbanity) at the organism scale (40 X 40 cm); Urb. local for the urbanity at the local scale (20 X 20 m); Urb. landscape for the urbanity at the landscape scale (3 X 3 km).

| **Internode space** | ***A. verlotiorum*** | ***E. canadensis*** | ***G. quadriradiata*** | ***M.discoidea*** | ***S. inaequidens*** | ***S. gigantea*** |
| --- | --- | --- | --- | --- | --- | --- |
| (Intercept) | ***1.81^***(***)^*** | *0.27* | ***1.39^***(***)^*** | ***-0.45^**(*)^*** | ***0.97^***(***)^*** | *0.14* |
|  | (0.46) | (0.67) | (0.18) | (0.17) | (0.24) | (0.17) |
| Urb. organism | ***-0.05^***(***)^*** | <-0.001 | <0.001 | *<-0.001* | ***-0.01^*(*)^*** | ***0.01^**(**)^*** |
|  | (0.01) | (<0.001) | (<0.001) | (<0.001) | (<0.001) | (<0.001) |
| Urb. local | *0.01* | <0.001 | <0.001 | <-0.001 | <0.001 |  |
|  | (<0.001) | (<0.001) | (<0.001) | (<0.001) | (<0.001) |  |
| Urb. landscape | *-0.02* | <-0.001 | <0.001 |  | <-0.001 | *0.01* |
|  | (0.01) | (0.01) | (<0.001) |  | (<0.001) | (<0.001) |
| SVF | -0.83 | *-0.65^(*)^* |  |  |  |  |
|  | (1.15) | (2.27) |  |  |  |  |
| SVF^2 | *-1.15^(**)^* | -0.33 |  |  |  |  |
|  | (1.06) | (1.91) |  |  |  |  |
| Vegetation cover | *-0.39* | 0.07 | -0.03 |  | -0.08 | *0.14* |
|  | (0.20) | (0.39) | (0.12) |  | (0.20) | (0.18) |
| Field season 2019 |  | *-0.28^(***)^* | ***-0.57^**(**)^*** |  | -0.13 | **-0.41^**(**)^** |
|  |  | (1.72) | (0.18) |  | (0.28) | (0.14) |
| Urb. landscape 🞫Urb. organism |  |  |  |  |  | *<-****0.001^**(**)^*** |
|  |  |  |  |  |  | (<0.001) |
| Urb. local 🞫 Urb. landscape | ***<-0.001^***(***)^*** |  |  |  |  |  |
|  | (<0.001) |  |  |  |  |  |
| Urb. organism 🞫 SVF |  | <-0.001 |  |  |  |  |
|  |  | (0.01) |  |  |  |  |
| Urb. local 🞫 Urb. organism | ***<0.001^***(***)^*** |  |  |  |  |  |
|  | (<0.001) |  |  |  |  |  |
| Urb. organism 🞫 Field season 2019 |  | <0.001 |  |  | <0.001 |  |
|  |  | (<0.001) |  |  | (<0.001) |  |
| Urb. landscape 🞫 Field season 2019 |  | <-0.001 |  |  |  | <-0.001 |
|  |  | (0.01) |  |  |  | (<0.001) |
| SVF^2 🞫 Urb. organism |  | <-0.001 |  |  |  |  |
|  |  | (<0.001) |  |  |  |  |
| Urb. landscape 🞫 SVF | 0.03 | <0.001 |  |  |  |  |
|  | (0.04) | (0.02) |  |  |  |  |
| SVF^2 🞫 Urb. landscape | *0.04^(**)^* |  |  |  |  |  |
|  | (0.04) |  |  |  |  |  |
| SVF 🞫 Field season 2019 |  | -2.48 |  |  |  |  |
|  |  | (6.37) |  |  |  |  |
| SVF^2 🞫 Field season 2019 |  | 2.26 |  |  |  |  |
|  |  | (5.63) |  |  |  |  |
| Urb. organism 🞫 Vegetation cover | ***0.04^**(**)^*** |  |  |  |  | ***-0.01^**(**)^*** |
|  | (0.01) |  |  |  |  | (<0.001) |
| Urb. local 🞫 Vegetation cover | ***-0.02^*(*)^*** |  |  |  |  |  |
|  | (0.01) |  |  |  |  |  |
| SVF 🞫 Vegetation cover |  | -0.15 |  |  |  |  |
|  |  | (0.70) |  |  |  |  |
| SVF^2 🞫 Vegetation cover |  | -0.07 |  |  |  |  |
|  |  | (0.44) |  |  |  |  |
| Vegetation cover 🞫 Field season 2019 |  | 0.01 |  |  |  |  |
|  |  | (0.10) |  |  |  |  |
| R² | 0.68 | 0.39 | 0.13 | 0.06 | 0.06 | 0.32 |
| Num. obs. | 54 | 112 | 72 | 40 | 74 | 93 |
|  | ^***^p < <0.0011; ^**^p < 0.01; ^*^p < 0.05 | | | | | |

**Appendix 7:** Averaged models information

Table 1: Information for the across-species averaged models (see Methods). Variables present in the models are coded as follows: 1 - Urbanity organism, 2 - SVF, 3 - SVF², 4 - Vegetation cover, 5 - Field season 2019, 6 - SVF 🞫 Field season 2019, 7 - SVF 🞫 Vegetation cover, 8 - Vegetation cover 🞫 Field season 2019, 9 - Winter temperatures, 10 - SVF 🞫 Winter temperatures.

|  | **Chlorophyll content** | **Flavonols index** | **Anthocyanins index** | **Specific leaf area** | **Plant height** | **Internode space** |
| --- | --- | --- | --- | --- | --- | --- |
| Num. obs. | 345 | 345 | 345 | 456 | 473 | 473 |
| Averaged model information | | | | | | |
| Number of models | 2 | 2 | 2 | 4 | 9 | 5 |
| Variables | 4,5,8 ; 4,5,8,10 | 2,4,5,7,9,10 ;2,3,5,7,9,10 | 2,5 ; 2,3 ; 2,3,5 | 2,3,5 ; 2,3,4,5,8 ; 2,4,5,8 ; 2,5 | 1,2,3,5 ; 1,2,3,5,6 ; 1,2,3,4,5,7 ; 1,3,5 ; 1,2,3,4,5,7,8 ; 1,5 ; 1,2,5 ; 1,2,3,4,5 ; 1,2,5,6 | 2,3,5,6 ; 5 ; 2,5,6 ; 2,3,5 ; 2,5 |
| df | 6 ; 7 | 9 ; 10 | 5 ; 5 ; 6 | 6 ; 8 ; 7 ; 5 | 7 ; 8 ; 9 ; 6 ; 10 ; 5 ; 6 ; 8 ; 7 | 7 ; 4 ; 6 ; 6 ; 5 |
| AICc | 765.5 ; 767.23 | -88.84 ; -88.36 | -442.00 ; -440.79 ; -440.03 | 626.55 ; 626.76 ; 627.37 ; 627.45 | 1026.15 ; 1026.53 ; 1026.99 ; 1027.16 ; 1027.32 ; 1027.33 ; 1027.35 ; 1027.60 ; 1027.97 | 617.67 ; 618.01 ; 618.38 ; 318.39 ; 619.01 |
| Model with the lowest AIC information | | | | | | |
| Num. groups : Species | 5 | 5 | 5 | 6 | 6 | 6 |
| Var: Species (Intercept) | 0.10 | 0.04 | 0.003 | 0.06 | 0.59 | 0.67 |
| Var: Residual | 0.49 | 0.04 | 0.01 | 0.21 | 0.45 | 0.46 |

Table 2: Information for the species-specific averaged models for the specific leaf area (SLA). Variables, if present in the models, are coded as follows: 1 - Urbanity organism, 2 - Urbanity local, 3 - Urbanity landscape, 4 - SVF, 5 - SVF², 6 - Vegetation cover, 7 - Field season 2019, 8 - Urbanity organism 🞫 Urbanity landscape, 9 - Urbanity organism 🞫 Urbanity local, 10 - Urbanity local 🞫 Urbanity landscape, 11 - Urbanity organism 🞫 SVF? 12 - Urbanity local 🞫 SVF, 13 - Urbanity landscape 🞫 SVF, 14 - Urbanity organism 🞫 SVF², 15 - Urbanity local 🞫 SVF², 16 - Urbanity landscape 🞫 SVF², 17 - Urbanity organism 🞫 Field season 2019, 18 - Urbanity local 🞫 Field season 2019, 19 - Urbanity landscape 🞫 Field season 2019, 20 - SVF 🞫 Field season 2019, 21 - SVF² 🞫 Field season 2019, 22 - Urbanity organism 🞫 Vegetation cover, 23 - Urbanity local 🞫 Vegetation cover, 24 - Urbanity landscape 🞫 Vegetation cover, 25 - SVF 🞫 Vegetation cover, 26 - SVF² 🞫 Vegetation cover, 27 - Vegetation cover 🞫 Field season 2019.

| **SLA** | *A. verlotiorum* N = 53 | | | *E. canadensis* N = 107 | | | *G. quadriradiata* N = 70 | | | *M. discoidea* N = 68 | | | | *S. inaequidens* N = 72 | | | *S. gigantea* N = 86 | | |
| --- | --- | --- | --- | --- | --- | --- | --- | --- | --- | --- | --- | --- | --- | --- | --- | --- | --- | --- | --- |
| *Number of models* | 13 | | | 41 | | | 19 | | | 41 | | | | 7 | | | 8 | | |
| *Model information* | Variables | df | AIC | Variables | df | AIC | Variables | df | AIC | | Variables | df | AIC | Variables | df | AIC | Variables | df | AIC |
|  | 4,5,7 | 5 | 5.02 | 1,2,3,5,7,10,16 | 9 | 66.18 | 3,6,7,27 | 6 | 183.93 | | 1,2,3,5,7,10,16 | 9 | 66.18 | 4,7 | 4 | 75.45 | 4,6,7,27 | 6 | 81.63 |
|  | 2,4,7 | 5 | 5.38 | 1,3,5,7,16,21 | 8 | 66.40 | 3,6,7,19,27 | 7 | 184.17 | | 1,3,5,7,16,21 | 8 | 66.40 | 4,5 | 4 | 75.61 | 5,6,7,27 | 6 | 81.87 |
|  | 2,4,5,7 | 6 | 6.07 | 1,2,3,5,7,10,16,21 | 10 | 66.66 | 3,6,7,24,27 | 7 | 184.40 | | 1,2,3,5,7,10,16,21 | 10 | 66.66 | 1,4,7 | 5 | 76.30 | 6,7,27 | 5 | 81.88 |
|  | 2,4,6,7 | 6 | 6.17 | 1,2,3,4,5,7,10,16,20,21 | 12 | 66.68 | 4,7 | 4 | 184.49 | | 1,2,3,5,4,7,10,16,20,21 | 12 | 66.68 | 1,4,5 | 5 | 76.44 | 3,4,6,7,27 | 7 | 82.48 |
|  | 4,7 | 4 | 6.17 | 1,3,5,7,16 | 7 | 66.69 | 3,4,6,7,27 | 7 | 184.900 | | 1,3,5,7,16 | 7 | 66.69 | 4,6,7 | 5 | 76.86 | 3,5,6,7,27 | 7 | 82.89 |
|  | 2,4,5,6,7 | 7 | 6.44 | 1,2,3,4,7,10,16 | 9 | 66.80 | 3,6,7,19,24,27 | 8 | 184.980 | | 2,3,5,4,7,10,13 | 9 | 66.80 | 4,5,6 | 5 | 76.95 | 3,6,7,27 | 6 | 83.29 |
|  | 4,5,6,7,26 | 7 | 6.55 | 1,2,3,5,7,16,21 | 9 | 66.80 | 3,4,6,19,24,27 | 8 | 185.000 | | 1,2,3,5,7,16,21 | 9 | 66.80 | 4,7,20 | 5 | 77.380 | 4,6,7,25,27 | 7 | 83.34 |
|  | 2,4,7,18 | 6 | 6.65 | 1,2,3,5,7,16 | 8 | 66.90 | 5,7 | 4 | 185.190 | | 1,2,3,5,7,16 | 8 | 66.90 |  |  |  | 1,3,4,6,7,8,27 | 9 | 83.35 |
|  | 2,5,7 | 5 | 6.67 | 1,3,5,6,7,16,21 | 9 | 66.91 | 3,5,6,7,27 | 7 | 185.320 | | 1,3,5,6,7,16,21 | 9 | 66.91 |  |  |  |  |  |  |
|  | 2,4,5,7,12 | 7 | 6.760 | 1,2,3,5,7,10,16,18 | 10 | 67.21 | 3,6,7,19 | 6 | 185.330 | | 1,2,3,5,7,10,16,18 | 10 | 67.21 |  |  |  |  |  |  |
|  | 2,4,5,7,15 | 7 | 6.820 | 1,3,4,5,6,7,16,20,21 | 12 | 67.24 | 3,6,7 | 5 | 185.380 | | 1,3,5,4,6,7,13,16,20,21 | 12 | 67.24 |  |  |  |  |  |  |
|  | 4,5,6,7,25 | 7 | 6.850 | 1,2,5,7,18 | 7 | 67.41 | 6,7,27 | 5 | 185.390 | | 1,2,5,7,18 | 7 | 67.41 |  |  |  |  |  |  |
|  | 2,3,4,7,8 | 7 | 6.980 | 1,2,3,4,5,7,10,16,20,21 | 12 | 67.46 | 4,6,7,27 | 6 | 185.450 | | 1,2,3,5,4,7,10,13,20,21 | 12 | 67.46 |  |  |  |  |  |  |
|  |  |  |  | 1,3,4,5,7,16,20,21 | 10 | 67.53 | 3,5,6,7,19,27 | 8 | 185.470 | | 1,3,5,4,7,16,21,20 | 10 | 67.53 |  |  |  |  |  |  |
|  |  |  |  | 1,5,7 | 5 | 67.54 | 3,4,5,6,7 | 7 | 185.580 | | 1,5,7 | 5 | 67.54 |  |  |  |  |  |  |
|  |  |  |  | 1,4,5,6,7,20,21,25,26 | 11 | 67.54 | 4,5,6,7 | 6 | 185.590 | | 1,4,5,6,7,20,21,25,26 | 11 | 67.54 |  |  |  |  |  |  |
|  |  |  |  | 1,2,3,4,5,7,16,20,21, | 11 | 67.59 | 4,5,7 | 5 | 185.650 | | 1,2,3,4,5,7,16,21,20 | 11 | 67.59 |  |  |  |  |  |  |
|  |  |  |  | 1,2,3,4,5,10,6,7,16,20,21 | 13 | 67.61 | 4,6,7 | 5 | 185.690 | | 1,2,3,4,5,6,7,10,16,20,21 | 13 | 67.61 |  |  |  |  |  |  |
|  |  |  |  | 1,3,4,5,6,7,16,20,21 | 11 | 67.61 | 5,6,7,27 | 6 | 185.760 | | 1,3,4,5,6,7,16,20,21 | 11 | 67.61 |  |  |  |  |  |  |
|  |  |  |  | 1,2,3,5,7,16,18 | 9 | 67.62 |  |  |  | | 1,2,3,5,7,16,18 | 9 | 67.62 |  |  |  |  |  |  |
|  |  |  |  | 1,3,5,6,7,16 | 8 | 67.64 |  |  |  | | 1,3,5,6,7,16 | 8 | 67.64 |  |  |  |  |  |  |
|  |  |  |  | 1,2,3,5,6,7,10,16 | 10 | 67.78 |  |  |  | | 1,2,3,5,6,7,10,16 | 10 | 67.78 |  |  |  |  |  |  |
|  |  |  |  | 1,2,3,4,7,10,16,21 | 10 | 67.79 |  |  |  | | 2,3,4,5,7,10,13,20 | 10 | 67.79 |  |  |  |  |  |  |
|  |  |  |  | 1,3,4,5,7,16,20,21 | 11 | 67.80 |  |  |  | | 1,3,5,4,7,13,16,20,21 | 11 | 67.80 |  |  |  |  |  |  |
|  |  |  |  | 1,2,3,5,7,16,18,21 | 10 | 67.81 |  |  |  | | 1,2,3,5,7,16,18,21 | 10 | 67.81 |  |  |  |  |  |  |
|  |  |  |  | 1,2,3,4,7,16 | 8 | 67.82 |  |  |  | | 2,3,4,5,7,13 | 8 | 67.82 |  |  |  |  |  |  |
|  |  |  |  | 1,2,4,7,18 | 7 | 67.85 |  |  |  | | 2,4,5,7,18 | 7 | 67.85 |  |  |  |  |  |  |
|  |  |  |  | 4,5,6,7,20,21,25,26 | 10 | 67.86 |  |  |  | | 4,5,6,7,20,21,25,26 | 10 | 67.86 |  |  |  |  |  |  |
|  |  |  |  | 1,5,7,21 | 6 | 67.92 |  |  |  | | 1,5,7,21 | 6 | 67.87 |  |  |  |  |  |  |
|  |  |  |  | 1,2,3,5,7,10,16,18,21 | 11 | 67.95 |  |  |  | | 1,2,3,5,7,10,16,18,21 | 11 | 67.92 |  |  |  |  |  |  |
|  |  |  |  | 1,2,5,7 | 6 | 68.02 |  |  |  | | 1,2,5,7 | 6 | 67.95 |  |  |  |  |  |  |
|  |  |  |  | 1,2,3,5,10,6,7,10,16,21 | 11 | 68.02 |  |  |  | | 1,2,3,5,6,7,10,16,21 | 11 | 68.02 |  |  |  |  |  |  |
|  |  |  |  | 1,3,4,7,16 | 7 | 68.02 |  |  |  | | 1,3,4,7,13 | 7 | 68.02 |  |  |  |  |  |  |
|  |  |  |  | 1,2,3,4,7,10,16,18 | 10 | 68.03 |  |  |  | | 1,2,3,4,7,10,18,13 | 10 | 68.03 |  |  |  |  |  |  |
|  |  |  |  | 1,2,3,5,7,10,16,19 | 10 | 68.05 |  |  |  | | 1,2,3,5,7,10,16,19 | 10 | 68.05 |  |  |  |  |  |  |
|  |  |  |  | 1,16,2,3,5,10,7,8 | 10 | 68.06 |  |  |  | | 1,2,3,5,7,8,10,16 | 10 | 68.06 |  |  |  |  |  |  |
|  |  |  |  | 1,4,5,6,7,20,21,26 | 10 | 68.07 |  |  |  | | 1,4,5,6,7,20,21,26 | 10 | 68.07 |  |  |  |  |  |  |
|  |  |  |  | 1,2,3,4,5,7,10,16,20,21 | 13 | 68.10 |  |  |  | | 1,2,3,5,4,7,10,13,16,20,21 | 13 | 68.10 |  |  |  |  |  |  |
|  |  |  |  | 1,4,7 | 5 | 68.10 |  |  |  | | 1,4,7 | 5 | 68.10 |  |  |  |  |  |  |
|  |  |  |  | 1,3,5,6,7,16,21,27 | 10 | 68.11 |  |  |  | | 1,3,5,6,7,16,21,27 | 10 | 68.11 |  |  |  |  |  |  |
|  |  |  |  | 1,2,3,4,7,10,11,16 | 10 | 68.11 |  |  |  | | 1,2,3,4,7,10,13,22 | 10 | 68.11 |  |  |  |  |  |  |

Table 3: Information for the species-specific averaged models for the anthocyanins index. Variables, if present in the models, are coded as follows: 1 - Urbanity organism, 2 - Urbanity local, 3 - Urbanity landscape, 4 - SVF, 5 - SVF², 6 - Vegetation cover, 7 - Field season 2019, 8 - Urbanity organism 🞫 Urbanity landscape, 9 - Urbanity organism 🞫 Urbanity local, 10 - Urbanity local 🞫 Urbanity landscape, 11 - Urbanity organism 🞫 SVF? 12 - Urbanity local 🞫 SVF, 13 - Urbanity landscape 🞫 SVF, 14 - Urbanity organism 🞫 SVF², 15 - Urbanity local 🞫 SVF², 16 - Urbanity landscape 🞫 SVF², 17 - Urbanity organism 🞫 Field season 2019, 18 - Urbanity local 🞫 Field season 2019, 19 - Urbanity landscape 🞫 Field season 2019, 20 - SVF 🞫 Field season 2019, 21 - SVF² 🞫 Field season 2019, 22 - Urbanity organism 🞫 Vegetation cover, 23 - Urbanity local 🞫 Vegetation cover, 24 - Urbanity landscape 🞫 Vegetation cover, 25 - SVF 🞫 Vegetation cover, 26 - SVF² 🞫 Vegetation cover, 27 - Vegetation cover 🞫 Field season 2019.

| **Anthocyanins** | *A. verlotiorum* N = 54 | | | *E. canadensis* N = 112 | | | *G. quadriradiata* N = 72 | | | *S. inaequidens* N = 68 | | | *S. gigantea* N = 75 | | |
| --- | --- | --- | --- | --- | --- | --- | --- | --- | --- | --- | --- | --- | --- | --- | --- |
| *Number of models* | 10 | | | 10 | | | 10 | | | 2 | | | 7 | | |
| *Model information* | Variables | df | AIC | Variables | df | AIC | Variables | df | AIC | Variables | df | AIC | Variables | df | AIC |
|  | 1, 4, 10 | 5 | -90.71 | 3, 4, 6, 12 | 6 | -151.26 | 1, 4, 6, 10 | 6 | -67.84 | 1, 2, 3, 4, 7, 9, 15 | 9 | -59.17 | 5, 6 | 4 | 118.02 |
|  | 1, 5, 18 | 5 | -90.70 | 3, 5, 6, 20 | 6 | -151.12 | 1, 4, 6 | 5 | -67.76 | 1, 2, 3, 4, 7, 15 | 8 | -57.39 | 4, 6 | 4 | 117.98 |
|  | 4 | 3 | -90.36 | 3, 4, 6 | 5 | -150.16 | 1, 6 | 4 | -67.74 |  |  |  | 1, 5, 6, 13 | 6 | 117.06 |
|  | 5.00 | 3 | -90.23 | 3, 6 | 4 | -150.09 | 4, 6 | 4 | -67.55 |  |  |  | 1, 4, 6, 13 | 6 | 117.00 |
|  | 1, 2, 6, 7, 13 | 7 | -89.92 | 3, 5, 6 | 5 | -150.00 | 1, 5, 6 | 5 | -67.40 |  |  |  | 1, 5, 6 | 5 | 116.71 |
|  | 1, 5 | 4 | -89.87 | 3, 4, 6, 12, 16 | 7 | -149.72 | 1, 5, 6, 18 | 6 | -67.26 |  |  |  | 1, 4, 6 | 5 | 116.55 |
|  | 1, 4 | 4 | -89.82 | 3, 5, 6, 17, 20 | 7 | -149.58 | 6.00 | 3 | -67.06 |  |  |  | 5, 6, 17 | 5 | 116.15 |
|  | 1 | 3 | -89.11 | 3, 4, 6, 12, 20 | 7 | -149.56 | 4, 5 | 4 | -66.97 |  |  |  |  |  |  |
|  | 1, 2, 5, 7, 18 | 7 | -89.07 | 3, 5, 6, 20 | 7 | -149.50 | 4, 5, 6 | 5 | -66.27 |  |  |  |  |  |  |
|  | 1, 2, 4, 7, 10 | 7 | -88.97 | 6 | 3 | -149.43 | 1, 4, 5, 6, 10 | 7 | -65.93 |  |  |  |  |  |  |
|  | Null | 2 | -88.79 | 3, 4, 6, 16 | 6 | -149.31 |  |  |  |  |  |  |  |  |  |
|  | 1, 2, 4, 6, 7, 13 | 8 | -88.78 |  |  |  |  |  |  |  |  |  |  |  |  |
|  | 1, 2, 5, 6, 7, 13 | 8 | -88.71 |  |  |  |  |  |  |  |  |  |  |  |  |

Table 4: Information for the species-specific averaged models for the flavonols index. Variables, if present in the models, are coded as follows: 1 - Urbanity organism, 2 - Urbanity local, 3 - Urbanity landscape, 4 - SVF, 5 - SVF², 6 - Vegetation cover, 7 - Field season 2019, 8 - Urbanity organism 🞫 Urbanity landscape, 9 - Urbanity organism 🞫 Urbanity local, 10 - Urbanity local 🞫 Urbanity landscape, 11 - Urbanity organism 🞫 SVF? 12 - Urbanity local 🞫 SVF, 13 - Urbanity landscape 🞫 SVF, 14 - Urbanity organism 🞫 SVF², 15 - Urbanity local 🞫 SVF², 16 - Urbanity landscape 🞫 SVF², 17 - Urbanity organism 🞫 Field season 2019, 18 - Urbanity local 🞫 Field season 2019, 19 - Urbanity landscape 🞫 Field season 2019, 20 - SVF 🞫 Field season 2019, 21 - SVF² 🞫 Field season 2019, 22 - Urbanity organism 🞫 Vegetation cover, 23 - Urbanity local 🞫 Vegetation cover, 24 - Urbanity landscape 🞫 Vegetation cover, 25 - SVF 🞫 Vegetation cover, 26 - SVF² 🞫 Vegetation cover, 27 - Vegetation cover 🞫 Field season 2019.

| **Flavonols** | *A. verlotiorum* N = 54 | | | *E. canadensis* N = 113 | | | *G. quadriradiata* N = 61 | | | *S. inaequidens* N = 63 | | | *S. gigantea* N = 77 | | |
| --- | --- | --- | --- | --- | --- | --- | --- | --- | --- | --- | --- | --- | --- | --- | --- |
| *Number of models* | 12 | | | 6 | | | 9 | | | 6 | | | 9 | | |
| *Model information* | Variables | df | AIC | Variables | df | AIC | Variables | df | AIC | Variables | df | AIC | Variables | df | AIC |
|  | 2,3,4,6,7,11,27 | 9 | -64.43 | 4,7 | 4 | -74.78 | 1,2,4,5,6,9,25 | 9 | -3.87 | 4,7 | 4 | 5.50 | 1,2,3,57,14,18,23 | 10 | -161.12 |
|  | 2,3,5,6,7,26,27 | 9 | -63.74 | 5,6 | 4 | -74.54 | 1,2,4,6,9,11,14,25 | 11 | -3.20 | 4,5 | 4 | 5.55 | 2,3,4,2,7,14,18,23 | 10 | -161.05 |
|  | 2,3,4,7,20 | 7 | -63.16 | 2,4,7 | 5 | -73.47 | 1,2,4,6,9,25 | 8 | -3.04 | 4,5,21 | 5 | 5.85 | 2,3,4,5,7,14,23 | 9 | -160.02 |
|  | 3,4,6,7,11,27 | 8 | -63.03 | 1,5,6 | 5 | -73.25 | 1,2,4,5,6,9,26 | 9 | -2.54 | 4,7,20 | 5 | 5.90 | 1,2,4,6,7,14,23 | 9 | -160.84 |
|  | 2,3,4,6,7,10,20 | 9 | -62.27 | 1,4,7 | 5 | -73.25 | 1,2,4,5,6,9,10,25 | 10 | -2.53 | 4,5,6 | 5 | 7.18 | 1,2,4,6,7,14,23,25 | 10 | -159.54 |
|  | 2,3,4,5,6,7,11,27 | 10 | -62.73 | 2,5,6 | 5 | -73.14 | 1,2,4,5,6,9,15,25 | 10 | -2.43 | 4,6,7 | 5 | 7.21 | 1,2,4,6,7,14,18,23,25 | 11 | -159.37 |
|  | 2,3,4,6,7,20 | 8 | -62.74 |  |  |  | 1,2,4,5,6,9,14,25 | 10 | -2.23 |  |  |  | 1,2,4,6,7,14,17,23 | 10 | -159.33 |
|  | 3,4,5,6,7,11,27 | 9 | -62.74 |  |  |  | 1,2,4,5,6,9,11,14,26 | 11 | -2.14 |  |  |  | 1,2,5,6,7,14,23,26 | 10 | -159.24 |
|  | 2,3,4,6,7,10,11,12 | 10 | -62.67 |  |  |  | 1,2,4,5,6,9,25,26 | 10 | -1.88 |  |  |  | 1,2,5,6,7,14,17,23 | 10 | -159.19 |
|  | 5,2,3,6,7,26,27 | 10 | -62.53 |  |  |  |  |  |  |  |  |  |  |  |  |
|  | 2,3,4,6,7,11,18,27 | 10 | -62.53 |  |  |  |  |  |  |  |  |  |  |  |  |
|  | 2,3,5,6,26 | 7 | -62.55 |  |  |  |  |  |  |  |  |  |  |  |  |

Table 5: Information for the species-specific averaged models for the plant height. Variables, if present in the models, are coded as follows: 1 - Urbanity organism, 2 - Urbanity local, 3 - Urbanity landscape, 4 - SVF, 5 - SVF², 6 - Vegetation cover, 7 - Field season 2019, 8 - Urbanity organism 🞫 Urbanity landscape, 9 - Urbanity organism 🞫 Urbanity local, 10 - Urbanity local 🞫 Urbanity landscape, 11 - Urbanity organism 🞫 SVF? 12 - Urbanity local 🞫 SVF, 13 - Urbanity landscape 🞫 SVF, 14 - Urbanity organism 🞫 SVF², 15 - Urbanity local 🞫 SVF², 16 - Urbanity landscape 🞫 SVF², 17 - Urbanity organism 🞫 Field season 2019, 18 - Urbanity local 🞫 Field season 2019, 19 - Urbanity landscape 🞫 Field season 2019, 20 - SVF 🞫 Field season 2019, 21 - SVF² 🞫 Field season 2019, 22 - Urbanity organism 🞫 Vegetation cover, 23 - Urbanity local 🞫 Vegetation cover, 24 - Urbanity landscape 🞫 Vegetation cover, 25 - SVF 🞫 Vegetation cover, 26 - SVF² 🞫 Vegetation cover, 27 - Vegetation cover 🞫 Field season 2019.

| **Height** | *A. verlotiorum* N = 54 | | | *E. canadensis* N = 113 | | | *G. quadriradiata* N = 72 | | | *M. discoidea* N = 69 | | | *S. inaequidens* N = 75 | | | *S. gigantea* N = 93 | | |
| --- | --- | --- | --- | --- | --- | --- | --- | --- | --- | --- | --- | --- | --- | --- | --- | --- | --- | --- |
| *Number of models* | 6 | | | 12 | | | 17 | | | 6 | | | 7 | | | 7 | | |
| *Model information* | Variables | df | AIC | Variables | df | AIC | Variables | df | AIC | Variables | df | AIC | Variables | df | AIC | Variables | df | AIC |
|  | 1,2,4,5,17,11,14 | 18 | 43.86 | 1,3,4,6,7,13,17 | 10 | 270.23 | 1,3,7,17 | 6 | 164.18 | 1, 2, 6, 14 | 6 | 64.21 | 1.00 | 3 | 147.48 | 1,6,7,22 | 6 | 169.44 |
|  | 1,2,4,5,7,17,11,14,20 | 28 | 44.02 | 1,3,4,6,7,13,24 | 18 | 270.54 | 6,7,27 | 5 | 164.22 | 1, 2, 6, 7, 14 | 7 | 65.90 | 1,7 | 4 | 148.33 | 1,6,22 | 5 | 170.20 |
|  | 1,2,4,5,6,7,17,11,14,25,26 | 12 | 44.47 | 3,4,6,7,13,24 | 8 | 270.86 | 1,7,17 | 5 | 164.33 | 1, 2, 4, 6, 14 | 7 | 65.97 | 1,3 | 4 | 148.64 | 1,2,6,7,22,23 | 8 | 170.44 |
|  | 1,2,4,5,6,7,17,11,14,22,25,26 | 13 | 45.52 | 1,7,17 | 5 | 271.22 | 3,7 | 4 | 164.43 | 1, 2, 4, 6, 7, 14 | 8 | 66.04 | 1,6 | 4 | 148.86 | 1,2,6,7,22 | 7 | 170.52 |
|  | 1,2,4,5,6,17,11,14,26 | 28 | 45.52 | 1,3,4,6,7,11,13,17,24 | 28 | 271.23 | 6,7 | 4 | 165.05 | 1, 2, 5, 6, 14 | 7 | 66.08 | 1,4,5,7,20,21 | 8 | 149.13 | 1,4,6,7,22 | 7 | 171.19 |
|  | 1,2,4,5,6,17,11,12,14,25,26 | 13 | 45.84 | 1,3,4,6,7,13,17,27,24 | 28 | 271.46 | 1,6,7,17 | 6 | 165.06 | 1 | 3 | 66.19 | 1,3,7 | 5 | 149.25 | 1,2,6,22 | 6 | 171.21 |
|  |  |  |  | 1,3,4,6,7,11,13,24 | 10 | 271.18 | 1,3,7,8,17 | 7 | 165.24 |  |  |  | 1,3,6 | 5 | 149.28 | 1,5,6,7,22 | 7 | 171.23 |
|  |  |  |  | 1,3,6,7,13,17 | 8 | 271.19 | 6 | 3 | 165.26 |  |  |  |  |  |  |  |  |  |
|  |  |  |  | 1,2,3,4,6,7,13,24 | 10 | 271.19 | 3,6,7,27 | 6 | 165.33 |  |  |  |  |  |  |  |  |  |
|  |  |  |  | 2,3,1,4,6,7,13,13,17 | 28 | 271.19 | 1,6,7,17,27 | 7 | 165.36 |  |  |  |  |  |  |  |  |  |
|  |  |  |  | 1,5,7,17 | 6 | 272.02 | 1,6,7,27 | 6 | 165.49 |  |  |  |  |  |  |  |  |  |
|  |  |  |  | 1,4,7,17 | 6 | 272.22 | 1,3,7 | 5 | 165.50 |  |  |  |  |  |  |  |  |  |
|  |  |  |  |  |  |  | 1,7 | 4 | 165.74 |  |  |  |  |  |  |  |  |  |
|  |  |  |  |  |  |  | 3,4,6,7,25,27 | 8 | 165.82 |  |  |  |  |  |  |  |  |  |
|  |  |  |  |  |  |  | 3,6,7 | 5 | 165.97 |  |  |  |  |  |  |  |  |  |
|  |  |  |  |  |  |  | 3,5,6,7,26,27 | 8 | 165.97 |  |  |  |  |  |  |  |  |  |
|  |  |  |  |  |  |  | 1,6,7 | 5 | 166.02 |  |  |  |  |  |  |  |  |  |

Table 6: Information for the species-specific averaged models for the chlorophyll content. Variables, if present in the models, are coded as follows: 1 - Urbanity organism, 2 - Urbanity local, 3 - Urbanity landscape, 4 - SVF, 5 - SVF², 6 - Vegetation cover, 7 - Field season 2019, 8 - Urbanity organism 🞫 Urbanity landscape, 9 - Urbanity organism 🞫 Urbanity local, 10 - Urbanity local 🞫 Urbanity landscape, 11 - Urbanity organism 🞫 SVF? 12 - Urbanity local 🞫 SVF, 13 - Urbanity landscape 🞫 SVF, 14 - Urbanity organism 🞫 SVF², 15 - Urbanity local 🞫 SVF², 16 - Urbanity landscape 🞫 SVF², 17 - Urbanity organism 🞫 Field season 2019, 18 - Urbanity local 🞫 Field season 2019, 19 - Urbanity landscape 🞫 Field season 2019, 20 - SVF 🞫 Field season 2019, 21 - SVF² 🞫 Field season 2019, 22 - Urbanity organism 🞫 Vegetation cover, 23 - Urbanity local 🞫 Vegetation cover, 24 - Urbanity landscape 🞫 Vegetation cover, 25 - SVF 🞫 Vegetation cover, 26 - SVF² 🞫 Vegetation cover, 27 - Vegetation cover 🞫 Field season 2019.

| **Chlorophyll** | *A. verlotiorum* N = 54 | | | *E. canadensis* N = 113 | | | *G. quadriradiata* N = 61 | | | | *S. inaequidens* N = 63 | | | *S. gigantea* N = 77 | | |
| --- | --- | --- | --- | --- | --- | --- | --- | --- | --- | --- | --- | --- | --- | --- | --- | --- |
| *Number of models* | 6 | | | 11 | | | 6 | | | | 5 | | | 15 | | |
| *Model information* | Variables | df | AIC | Variables | df | AIC | | Variables | df | AIC | Variables | df | AIC | Variables | df | AIC |
|  | 2,7,18 | 5 | 109.23 | 3,5,7,16 | 6 | 205.44 | | 1,4,7,11,17 | 7 | 119.16 | Null | 2 | 147.96 | 1,2,3,4,6,7,8,14,19,23,27 | 14 | 145.76 |
|  | Null | 2 | 109.91 | 3,4,7,13 | 6 | 205.71 | | 1,5,7,14,17 | 7 | 119.63 | 2 | 3 | 148.95 | 2,3,6,7,18,19,23,24 | 10 | 146.14 |
|  | 7 | 3 | 110.27 | 3,4,5,7,16 | 7 | 206.48 | | 1,2,4,7,11,17 | 8 | 119.89 | 3 | 3 | 149.12 | 1,3,6,7,8,11,19,24,27 | 12 | 143.64 |
|  | 6,7,27 | 5 | 110.36 | 3,4,5,7,13 | 7 | 206.89 | | 1,2,5,7,14,17 | 8 | 119.99 | 5 | 3 | 149.64 | 2,3,6,7,18,19,23 | 9 | 146.38 |
|  | 2,5, 7,18, 28 | 7 | 110.72 | 3,5,7,16,21 | 7 | 207.04 | | 1,3,4,7,11,17 | 8 | 120.71 | 4 | 3 | 149.71 | 2,3,5,6,7,18,19,23,24 | 11 | 147.02 |
|  | 2, 4, 7,18, 21 | 7 | 111.11 | 3,5,7,16,19 | 7 | 207.14 | | 1,2,3,4,7,11,17 | 9 | 120.82 |  |  |  | 2,3,5,6,7,18,19,23,26 | 11 | 147.09 |
|  |  |  |  | 1,3,5,7,16 | 7 | 207.16 | |  |  |  |  |  |  | 1,3,6,7,8,11,17,19,24,27 | 11 | 147.22 |
|  |  |  |  | 2,3,5,7,16 | 7 | 207.19 | |  |  |  |  |  |  | 1,2,3,4,6,7,8,14,17,19,23,27 | 13 | 147.39 |
|  |  |  |  | 3,4,7,16,19 | 7 | 207.32 | |  |  |  |  |  |  | 2,3,5,6,7,18,19,23,24,26 | 15 | 147.43 |
|  |  |  |  | 3,5,6,7,16 | 7 | 207.33 | |  |  |  |  |  |  | 1,3,5,6,7,8,11,19,24,27 | 12 | 147.53 |
|  |  |  |  | 2,3,4,7,13 | 7.00 | 207.36 | |  |  |  |  |  |  | 2,3,5,6,7,18,19,23 | 10 | 147.54 |
|  |  |  |  |  |  |  | |  |  |  |  |  |  | 2,3,5,6,7,18,19,23,24,26 | 11 | 147.58 |
|  |  |  |  |  |  |  | |  |  |  |  |  |  | 2,3,5,6,7,18,19,23,26 | 10 | 147.60 |
|  |  |  |  |  |  |  | |  |  |  |  |  |  | 2,3,5,6,7,10,18,19,23,24,26 | 15 | 147.74 |
|  |  |  |  |  |  |  | |  |  |  |  |  |  | 1,2,3,4,6,7,8,14,19,24,27 | 13 | 147.75 |

Table 7: Information for the species-specific averaged models for the internode space. Variables, if present in the models, are coded as follows: 1 - Urbanity organism, 2 - Urbanity local, 3 - Urbanity landscape, 4 - SVF, 5 - SVF², 6 - Vegetation cover, 7 - Field season 2019, 8 - Urbanity organism 🞫 Urbanity landscape, 9 - Urbanity organism 🞫 Urbanity local, 10 - Urbanity local 🞫 Urbanity landscape, 11 - Urbanity organism 🞫 SVF? 12 - Urbanity local 🞫 SVF, 13 - Urbanity landscape 🞫 SVF, 14 - Urbanity organism 🞫 SVF², 15 - Urbanity local 🞫 SVF², 16 - Urbanity landscape 🞫 SVF², 17 - Urbanity organism 🞫 Field season 2019, 18 - Urbanity local 🞫 Field season 2019, 19 - Urbanity landscape 🞫 Field season 2019, 20 - SVF 🞫 Field season 2019, 21 - SVF² 🞫 Field season 2019, 22 - Urbanity organism 🞫 Vegetation cover, 23 - Urbanity local 🞫 Vegetation cover, 24 - Urbanity landscape 🞫 Vegetation cover, 25 - SVF 🞫 Vegetation cover, 26 - SVF² 🞫 Vegetation cover, 27 - Vegetation cover 🞫 Field season 2019.

| **Internode space** | *A. verlotiorum* N = 54 | | | *E. canadensis* N = 113 | | | *G. quadriradiata* N = 72 | | | *M. discoidea* N = 69 | | | *S. inaequidens* N = 75 | | | *S. gigantea* N = 93 | | |
| --- | --- | --- | --- | --- | --- | --- | --- | --- | --- | --- | --- | --- | --- | --- | --- | --- | --- | --- |
| *Number of models* | 2 | | | 29 | | | 4 | | | 8 | | | 9 | | | 2 | | |
| *Model information* | Variables | df | AIC | Variables | df | AIC | Variables | df | AIC | Variables | df | AIC | Variables | df | AIC | Variables | df | AIC |
|  | 1,2,3,5,6,9,10,16,22,23 | 12 | 62.87 | 5,7 | 4 | 222.31 | 6 | 3 | 168.37 | 1 | 3 | 136.21 | 1 | 3 | 168.40 | 1,3,6,7,8,22 | 8 | 132.73 |
|  | 1,2,3,4,6,9,10,13,22,23 | 12 | 63.74 | 1,4,7 | 5 | 222.56 | 3, 6 | 4 | 169.85 | 1,2 | 4 | 137.13 | 1,2 | 4 | 169.01 | 1,3,6,7,8,19,22 | 9 | 134.33 |
|  |  |  |  | 5,7 | 4 | 223.11 | 1, 6 | 4 | 169.89 | 1,7 | 4 | 137.79 | 1,7,17 | 5 | 169.09 |  |  |  |
|  |  |  |  | 1,5,7 | 5 | 223.26 | 2, 6 | 4 | 169.95 | 1,6,7,27 | 6 | 137.97 | 1,6 | 4 | 169.30 |  |  |  |
|  |  |  |  | 4,5,7,21,20 | 7 | 223.30 |  |  |  | 1,3 | 4 | 137.98 | 1,3 | 4 | 169.98 |  |  |  |
|  |  |  |  | 1,4,7,11 | 6 | 223.40 |  |  |  | 1,3,8 | 5 | 138.00 | 1,6 | 4 | 170.04 |  |  |  |
|  |  |  |  | 4,5,7 | 5 | 223.47 |  |  |  | 1,4 | 4 | 138.08 | 1,2,3 | 5 | 170.11 |  |  |  |
|  |  |  |  | 4,5,6,7 | 6 | 223.62 |  |  |  | 1,5 | 4 | 138.13 | 1,3,6 | 5 | 170.14 |  |  |  |
|  |  |  |  | 3,4,5,7,13,17,19 | 9 | 223.72 |  |  |  |  |  |  | 1,2,6,17 | 6 | 170.34 |  |  |  |
|  |  |  |  | 1,4,7,17 | 6 | 223.73 |  |  |  |  |  |  |  |  |  |  |  |  |
|  |  |  |  | 3,4,5,7 | 6 | 223.79 |  |  |  |  |  |  |  |  |  |  |  |  |
|  |  |  |  | 4,6,7 | 5 | 223.80 |  |  |  |  |  |  |  |  |  |  |  |  |
|  |  |  |  | 1,4,5,7,21,20 | 8 | 223.84 |  |  |  |  |  |  |  |  |  |  |  |  |
|  |  |  |  | 1,6,7 | 5 | 223.85 |  |  |  |  |  |  |  |  |  |  |  |  |
|  |  |  |  | 1,4,5,6,7,21,25,20 | 10 | 223.89 |  |  |  |  |  |  |  |  |  |  |  |  |
|  |  |  |  | 1,4,5,6,7,20,21,26 | 10 | 223.90 |  |  |  |  |  |  |  |  |  |  |  |  |
|  |  |  |  | 3,4,5,7,11,13,17,19 | 10 | 223.92 |  |  |  |  |  |  |  |  |  |  |  |  |
|  |  |  |  | 1,3,4,7,17,19 | 8 | 223.97 |  |  |  |  |  |  |  |  |  |  |  |  |
|  |  |  |  | 1,3,4,7,13,19 | 8 | 223.98 |  |  |  |  |  |  |  |  |  |  |  |  |
|  |  |  |  | 5,7,21 | 5 | 223.99 |  |  |  |  |  |  |  |  |  |  |  |  |
|  |  |  |  | 1,5,7,8 | 6 | 224.00 |  |  |  |  |  |  |  |  |  |  |  |  |
|  |  |  |  | 1,4,5,7 | 6 | 224.10 |  |  |  |  |  |  |  |  |  |  |  |  |
|  |  |  |  | 1,4,6,7,11 | 7 | 224.170 |  |  |  |  |  |  |  |  |  |  |  |  |
|  |  |  |  | 1,3,4,7,19 | 7 | 224.190 |  |  |  |  |  |  |  |  |  |  |  |  |
|  |  |  |  | 1,4,7,11,17 | 7 | 224.19 |  |  |  |  |  |  |  |  |  |  |  |  |
|  |  |  |  | 3,4,7 | 5 | 224.20 |  |  |  |  |  |  |  |  |  |  |  |  |
|  |  |  |  | 1,5,6,7 | 6 | 224.20 |  |  |  |  |  |  |  |  |  |  |  |  |
|  |  |  |  | 1,4,5,6,7,20,21,25,27 | 11 | 224.29 |  |  |  |  |  |  |  |  |  |  |  |  |
|  |  |  |  | 1,2,4,7 | 6 | 224.31 |  |  |  |  |  |  |  |  |  |  |  |  |
